# Toggling Stereochemical Activity through Interstitial Positioning of Cations between 2D V_2_O_5_ Double Layers

**DOI:** 10.1021/acs.chemmater.3c01463

**Published:** 2023-08-31

**Authors:** George Agbeworvi, Wasif Zaheer, Joseph V. Handy, Justin L. Andrews, Saul Perez-Beltran, Cherno Jaye, Conan Weiland, Daniel A. Fischer, Perla B. Balbuena, Sarbajit Banerjee

**Affiliations:** † Department of Chemistry and Department of Material Science and Engineering, Texas A&M University, College Station, Texas 77843, United States; ‡ Department of Chemical Engineering, Texas A&M University, College Station, Texas 77843, United States; § Material Measurement Laboratory, National Institute of Standards and Technology, Gaithersburg, Maryland 20899, United States

## Abstract

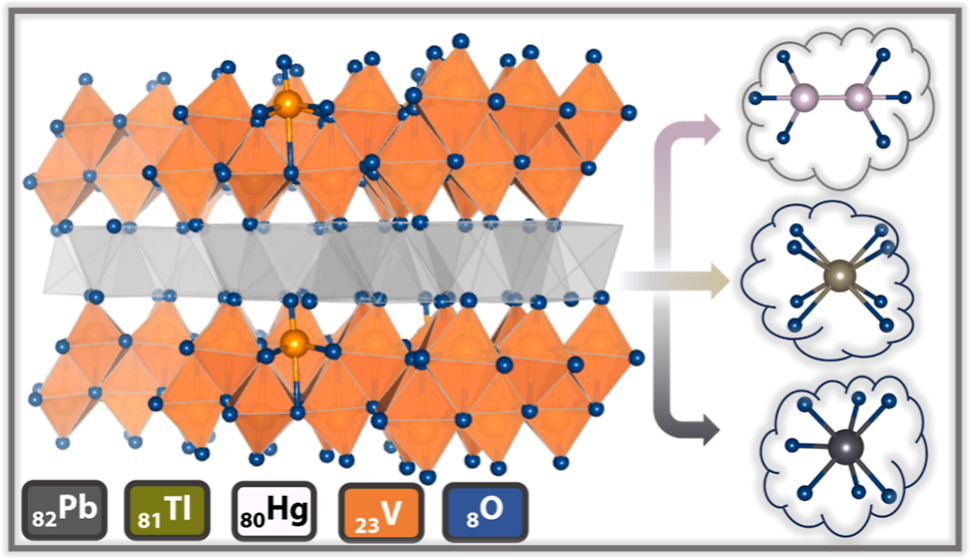

The 5/6s^2^ lone-pair electrons of p-block cations in their lower oxidation states are a versatile electronic and geometric structure motif that can underpin lattice anharmonicity and often engender electronic and structural instabilities that underpin the function of active elements in nonlinear optics, thermochromics, thermoelectrics, neuromorphic computing, and photocatalysis. In contrast to periodic solids where lone-pair-bearing cations are part of the structural framework, installing lone-pair-bearing cations in the interstitial sites of intercalation hosts provides a means of a systematically modulating electronic structure through the choice of the group and the period of the inserted cation while preserving the overall framework connectivity. The extent of stereochemical activity and the energy positioning of lone-pair-derived mid-gap states depend on the cation identity, stoichiometry, and strength of anion hybridization. V_2_O_5_ polymorphs are versatile insertion hosts that can accommodate a broad range of s-, p-, and d-block cations. However, the insertion of lone-pair-bearing cations remains largely underexplored. In this article, we examine the implications of varying the 6s^2^ cations situated in interlayer sites between condensed [V_4_O_10_]_*n*_ double layers. Systematic modulations of lattice distortions, electronic structure, and magnetic ordering are observed with increasing strength of stereochemical activity from group 12 to group 14 cations. We compare and contrast p-block-layered M_*x*_V_2_O_5_ (M = Hg, Tl, and Pb) compounds and map the significance of local off-centering arising from the stereochemical activity of lone-pair cations to the emergence of filled antibonding lone-pair 6s^2^–O 2p-hybridized mid-gap states mediated by second-order Jahn–Teller distortions. Crystallographic studies of cation coordination environments and the resulting modulation of V–V interactions have been used in conjunction with variable-energy hard X-ray photoelectron spectroscopy measurements, first-principles electronic structure calculations, and crystal orbital Hamilton population analyses to decipher the origins of stereochemical activity. Magnetic susceptibility measurements reveal antiferromagnetic signatures for all the three compounds. However, the differences in V–V interactions significantly affect the energy balance of the superexchange interactions, resulting in an ordering temperature of 160 and 260 K for Hg_0.5_V_2_O_5_ and δ-Tl_0.5_V_2_O_5_, respectively, as compared to 7 K for δ-Pb_0.5_V_2_O_5_. In δ-Pb_0.5_V_2_O_5_, the strong stereochemical activity of electron lone pairs and the resulting electrostatic repulsions enforce superlattice ordering, which strongly modifies the electronic localization patterns along the [V_4_O_10_] slabs, resulting in disrupted magnetic ordering and an anomalously low ordering temperature. The results demonstrate a versatile strategy for toggling the stereochemical activity of electron lone pairs to modify the electronic structure near the Fermi level and to mediate superexchange interactions.

## Introduction

Post-transition-metal 5/6s^2^ electron lone pairs of p-block cations in their lower oxidation states represent an attractive electronic structure motif that can be toggled between stereochemically active^1,2^ and inert end members based on the strength of anion hybridization. In periodic solids, the stereochemical activity of the lone pairs provides a means to realize anharmonic lattice distortions, drive electronic and structural instabilities, and evince redox-active filled mid-gap states at the Fermi level.^1,3−5^ Both the extent of lattice distortion and the energy positioning and dispersion of mid-gap states are tunable based on cation identity, interatomic separation, strength of anion hybridization, and stoichiometry of p-block cations.^6−8^ By some accounts, stereochemically active electron lone pairs occupy a volume equivalent to that of a fluoride ion.^9,10^ Lattice distortions resulting from lone-pair repulsions drive cation off-centering and lattice instabilities, which form the functional basis for applications in thermochromics, nonlinear optics, thermoelectrics, brain-inspired computing, and solar photocatalysis.^3,5,11−13^

The rich diversity of structural motifs observed in the binary vanadium–oxygen system is a result of the versatile connectivity of VO_*x*_ tetrahedra, square pyramids, and octahedra to connect through various combinations of edge- and corner-sharing connections.^14^ Consequently, the known polymorphs of V_2_O_5_ often adopt low-dimensional arrangements with an abundance of interstitial sites that can accommodate alkali, alkaline, transition metal, and even p-block cations.^15^ Installing low-valent p-block cations with stereochemically active lone pairs in the interstitial sites of V_2_O_5_ polymorphs provides a means of tuning lattice distortions and modulating electronic structure.^3,6,7^ As an example, replacing Pb^2+^ cations with Sn^2+^ cations in a tunnel-structured ternary vanadium oxide (β-M_*x*_V_2_O_5_) improved the overlap of lone-pair-derived mid-gap states with valence band edges of CdS and CdSe quantum dots, enabling ultrafast hole extraction.^16^ In contrast to compounds where lone-pair-bearing cations are part of the structural framework, installing p-block cations in interstitial sites provides a means of systematically modulating the electronic structure of the parent vanadium oxide structure through the choice of the group and the period of the inserted cation while preserving the overall structural connectivity. In this article, we examine the implications of varying the 6s^2^ cations situated in interlayer sites between condensed [V_4_O_10_]_*n*_ double layers for lattice distortions, electronic structure, and magnetic ordering. The stereochemical activity of the 6s^2^ lone pairs has a profound effect on the energetics of mid-gap states as mapped through hard X-ray photoemission spectroscopy (HAXPES) measurements and crystal orbital Hamiltonian parameter (COHP) analyses, as well as on the magnetic ordering of electron spins arrayed across the [V_4_O_10_] layers.

Across different V_2_O_5_ polymorphs, the stoichiometry of the interstitially positioned cations, the specific interstitial sites occupied, and the cation identity in M_*x*_V_2_O_5_ governs the localization and charge/spin ordering of electron density along the V_2_O_5_ framework.^17^ 2D M_*x*_V_2_O_5_ phases with intercalated cations residing between condensed double-layered [V_4_O_10_] sheets afford considerable tunability both in terms of the scope of cations and their solvation.^18^ This class of compounds exhibits a variety of distinctive properties including metal–insulator transitions (MITs), superconductivity, spin gaps, and charge ordering,^18−22^ which are governed by the relative separation, ordering, and stoichiometry of inserted cations. Vanadium 3d^1^ (*S* = 1/2) sites are stabilized upon cation insertion in the galleries between [V_4_O_10_] layers^18,19,23−25^ and show various low-dimensional ordering motifs including zigzag chains, ladders, and dimers.^21−26^

As illustrative examples of the rich coupling of spin, charge, orbital, and atomic degrees of freedom in these materials, at low temperatures, δ-M_*x*_V_2_O_5_ (M = Na and Sr) compounds exhibit a low-dimensional, spin-1/2 magnetism, and spin-gap characteristic. δ-Sr_0.5_V_2_O_5_ manifests strongly antiferromagnetic (AFM) ordering, which has been ascribed to the alternating exchange chain derived from ordering of nominally tetravalent and pentavalent vanadium centers. In contrast, the AFM behavior of δ-Na_0.5_V_2_O_5_ is characterized by large exchange coupling as a result of greater electron delocalization.^22^ Magnetic studies of δ-Ag_*x*_V_2_O_5_ (0.65 ≤ *x* ≤ 0.90) reveal a strong dependence on the cation stoichiometry, *x*. At low values of *x* (0.65–0.75), first-order structural transitions are observed at 220 K, concomitant with a magnetic transition. With increasing cation stoichiometry, *x* ∼ 0.80–0.85, a magnetic transition is instead observed at 170 K; the transition temperature is monotonically decreased upon further increasing *x*. Ultimately, linear chain behavior with low-temperature superexchange is observed at *x* ∼ 0.90.^26^ Despite the rich interplay between cation identity (M) and stoichiometry (*x*) in M_*x*_V_2_O_5_ bronzes, the insertion of p-block cations with stereochemically active lone pairs between 2D infinite [V_4_O_10_] layers remains almost entirely unexplored. In this work, we examine a series of double-layered M_*x*_V_2_O_5_ (M = Hg, Tl, and Pb) compounds and unravel the role of the lone pairs in inducing structural distortions, mediating magnetic ordering, and modulating the energy positioning of mid-gap states.

We demonstrate that lone-pair repulsions and concomitant lattice anharmonicity drive anisotropic structural distortions, which in turn modify the superexchange and electron correlation. Magnetic ordering temperatures of 160, 260, and 7 K are observed for Hg_0.5_V_2_O_5_, δ-Tl_0.5_V_2_O_5_, and δ-Pb_0.5_V_2_O_5_, respectively. Using a combination of structure elucidation from X-ray diffraction (XRD), HAXPES mapping of the orbital characteristic of valence band states, first-principles calculations of the electronic structure, and magnetic measurements, we examine the role of 6s^2^ lone pairs of Hg_2_^2+^, Tl^+^, and Pb^2+^ cations in modulating lattice distortions, mid-gap states at the valence band edge, and magnetic ordering.

## Results and Discussion

### Structure Elucidation and Coordination Environments

**Figure 1:**
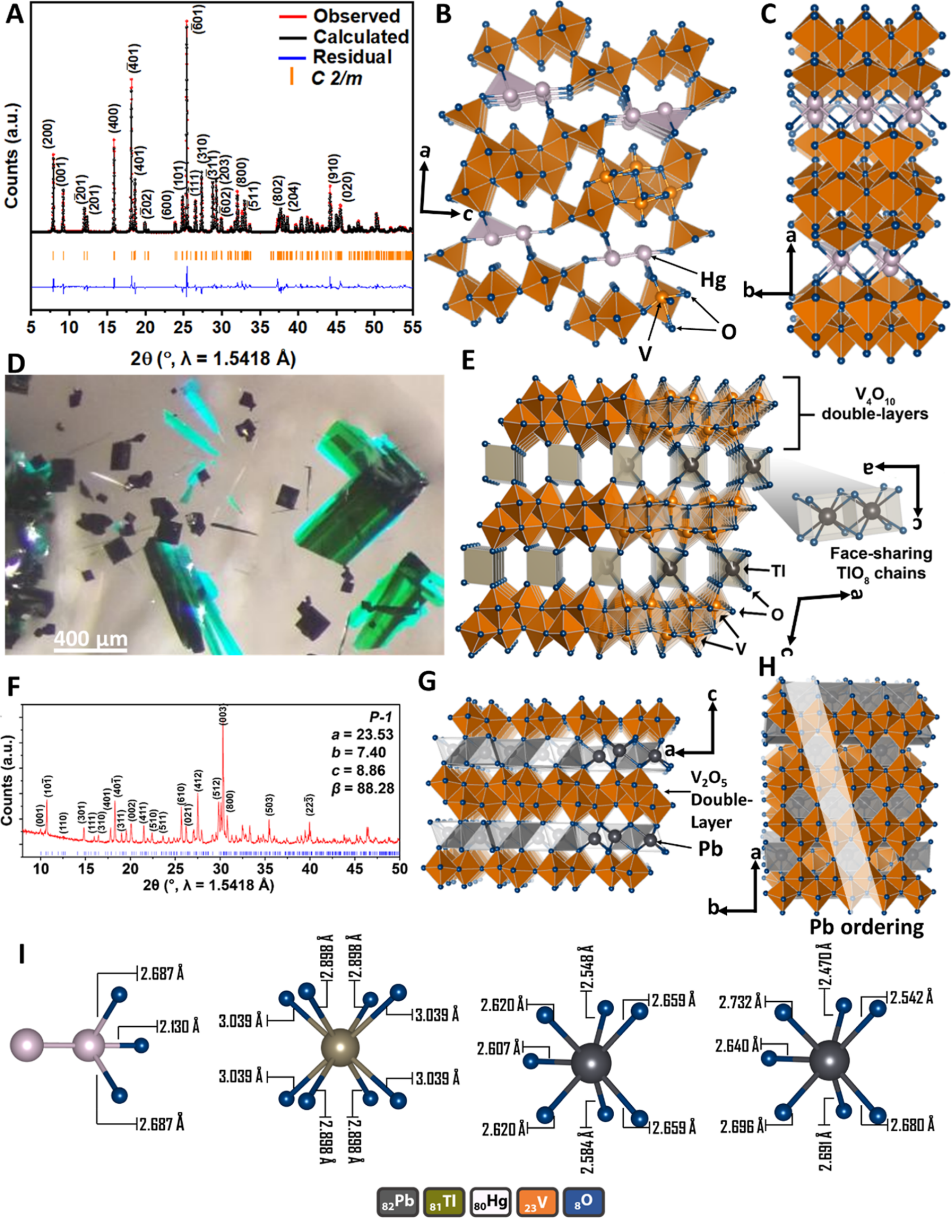
Structure elucidation. (A) Powder XRD pattern and (B) structure solution of Hg_*x*_V_2_O_5_. Details of Rietveld refinements are provided in Tables S1 and S2. (C) Perspective view illustrating the layered structure of (B) where the [V_4_O_10_] layers are separated by dimeric Hg_2_^2+^ cations. (D) Optical microscopy image of δ-Tl_*x*_V_2_O_5_ single crystals. (E) Structure solution obtained from the refinement of single-crystal XRD data of δ-Tl_*x*_V_2_O_5_. (F) Powder XRD pattern and (G) structure solution of δ-Pb_*x*_V_2_O_5_. Details of Rietveld refinements are provided in Tables S3 and S4. (H) The ordering of Pb^2+^ ions residing on the ab plane that gives rise to the 2 × 2 × 1 supercell is indicated using a clear white stripe. (I) Comparison of (left) Hg–O, (middle) Tl–O, and (right) Pb(1, 2)–O coordination illustrating the effect of stereochemical activity of 6s^2^ lone pairs in the latter.

Layered M_*x*_V_2_O_5_ (M = Hg, Tl, and Pb) have been prepared as described in the Experimental Section. Figure 1A shows the powder XRD pattern obtained for Hg_0.5_V_2_O_5_ at 295 K. The structure solution for this compound obtained from Rietveld refinement of the powder XRD data corresponds to a monoclinic *C*2/*m* space group with four crystallographically inequivalent vanadium centers as shown in Figure 1B (Tables S1 and S2). Figure 1C illustrates the layered framework where infinite [V_4_O_10_] slabs are separated by dimeric Hg_2_^2+^ cations exhibiting a Hg–Hg bond distance of 2.51(3) Å (consistent with the 2.55(11) Å Hg–Hg interaction in Hg_2_Cl_2_).^27,28^ The mercury atoms are symmetry-related by an inversion center and constitute a Hg_2_^2+^ dumbbell with a symmetric oxygen environment. Three of the four vanadium atoms (V1–V3) are octahedrally coordinated, whereas the fourth vanadium atom, V4, resides at the center of a distorted trigonal bipyramid. The VO_6_ octahedra form an edge-sharing network, whereas the VO_5_ trigonal bipyramids form a corner-sharing network (Figure 1B) that connects adjacent octahedral clusters. The as-synthesized Hg_0.5_V_2_O_5_ particles have rectangular cross-sections with lateral dimensions of ca. 5 μm and span hundreds of microns in length (Figure S1A). The energy-dispersive X-ray spectral map shown in Figure S1B indicates a homogeneous distribution of Hg, V, and O. EDX yields a Hg stoichiometry of *x* = 0.51, which is consistent with the 0.50 value determined by structural refinement. Several attempts to synthesize single crystals of Hg_*x*_V_2_O_5_ by melt quenching were unsuccessful likely because of the volatility of Hg.

Scanning electron microscopy (SEM) and transition electron microscopy (TEM) images in Figure S1C,E provide the evidence of the growth of single-crystalline nanowires of δ-Tl_0.50_V_2_O_5_. The nanowires are several hundred microns in length and 162 ± 88 nm in their lateral dimensions. An indexed SAED pattern with a high-resolution TEM image is shown in Figure S1F and attests to the single-crystalline nature of the nanowires. Powders of this compound are congruently melted, which enables the growth of single crystals as described in the Experimental Section. The crystal structure of δ-Tl_0.50_V_2_O_5_ from the single-crystal refinement is indexed to a monoclinic *C*2/*m* space group (Tables S5–S8). The crystal structure shown in Figure 1E illustrates the quasi-2D layered framework with Tl^+^ cations separating the infinite [V_4_O_10_] double layers. This layered motif is reflected in the plate-like habit of the single crystals shown in Figure 1D. The structure comprises 1D face-sharing chains of cubic TlO_8_ polyhedra and condensed [V_4_O_10_] double layers that extend infinitely in the *ab* crystallographic plane. The TlO_8_ cubic polyhedra play a pillaring role but are markedly different from the dimeric Hg_2_^2+^ ions in Figure 1B (note that the latter establishes 3D connectivity by disrupting and puckering the 2D structure and as such is not formally the δ-phase, although it has similar connectivity). Two crystallographically inequivalent vanadium atoms can be differentiated and reside at the centers of the distorted octahedra. The V(1)O_6_ and V(2)O_6_ octahedra share edges and corners to form the [V_4_O_10_] double layers extending along the *ab* planes. The Tl stoichiometry has been confirmed by EDS measurements shown in Figure S1D. Crystallographic information files corresponding to δ-Tl_0.5_V_2_O_5_ have been deposited in the Cambridge Structural Database and are available for access with deposition number 2170921.

Phase-pure and congruently melting δ-Pb_0.50_V_2_O_5_ powder and single crystals have been similarly synthesized as described in the experimental section. However, the single crystals were severely twinned, and as a result, structure solutions reported here are based on powder XRD. The powder XRD pattern is refined to the structure shown in Figure 1G, which indicates that the compound crystallizes in the triclinic *P*1̅ space group. Infinite [V_4_O_10_] double-layered slabs (Tables S3 and S4) are once again the primary structural motif but with an intriguing supercell. Six crystallographically inequivalent vanadium atoms can be differentiated, labeled V1–V6, and reside at the centers of the distorted octahedra. Edge- and corner-shared VO_6_ octahedra form infinite [V_4_O_10_] slabs that are separated by intercalated Pb cations. The Pb ions are coordinated by apical oxygens from the vanadium-centered octahedra. The Pb ions are situated at the center of a trigonal prismatic local coordination environment analogous to that previously reported for isostructural δ-Sr_0.5_V_2_O_5_^29^ and δ-Ag_0.85_V_2_O_5_.^26^ The supercell shown in Figure 1H reflects the ordering of Pb cations between the double layers, which in turns suggests a high degree of charge ordering across the [V_4_O_10_] slabs. The Pb stoichiometry has been confirmed by EDS measurements (Figure S2B). Figures S2A shows the SEM image of δ-Pb_0.50_V_2_O_5_ powders, which crystallize with rectangular cross-sections.

While the infinite 2D [V_4_O_10_] slabs are a recurrent structural motif across all the three compounds, the local coordination environments of the pillaring cations show distinct differences. The local coordination environments of Hg^+^, Tl^+^, and Pb^2+^ ions are sketched in Figure 1I. The Hg^+^ (one-half of the dumb-bell-shaped Hg_2_^2+^ dimer), Tl^+^, and Pb^2+^ ions are coordinated to three, eight, and six oxygen atoms, respectively. The Hg–O bond distances in Hg_0.5_V_2_O_5_ vary from 2.130 to 2.687 Å (average 2.501 Å); the Hg_2_^2+^ dimers severely distort the [V_4_O_10_] slabs. In δ-Tl_0.50_V_2_O_5_, the Tl–O bond distances vary from 2.898 to 3.039 Å (average 2.969 Å), whereas in δ-Pb_0.50_V_2_O_5_, the Pb–O bond distances range from 2.470 to 2.696 Å (average 2.620 Å). The stereochemical activity of the electron lone pairs of Pb atoms, which is examined in detail in subsequent sections, underpins the distinctive superlattice ordering pattern observed in δ-Pb_0.50_V_2_O_5_. Pb 6s^2^ states are closely matched in energy to O 2p states, which results in strong Pb–O hybridization manifested as a short Pb(2)–O(6) bond (2.47(9) Å). The filled antibonding states from this interaction hybridize with empty Pb 6p states to yield the lone-pair-derived states at the top of the valence band. The strong anchoring of Pb ions to specific oxygen atoms in the V_2_O_5_ layers, the resulting off-centering of Pb within its coordination environment, and the electrostatic repulsions between lone pairs underpins the disposition of Pb ions within the layers and the observed superlattice ordering.

### Electronic Structure and Stereochemical (In)activity of 6s^2^ Lone Pairs

**Figure 2:**
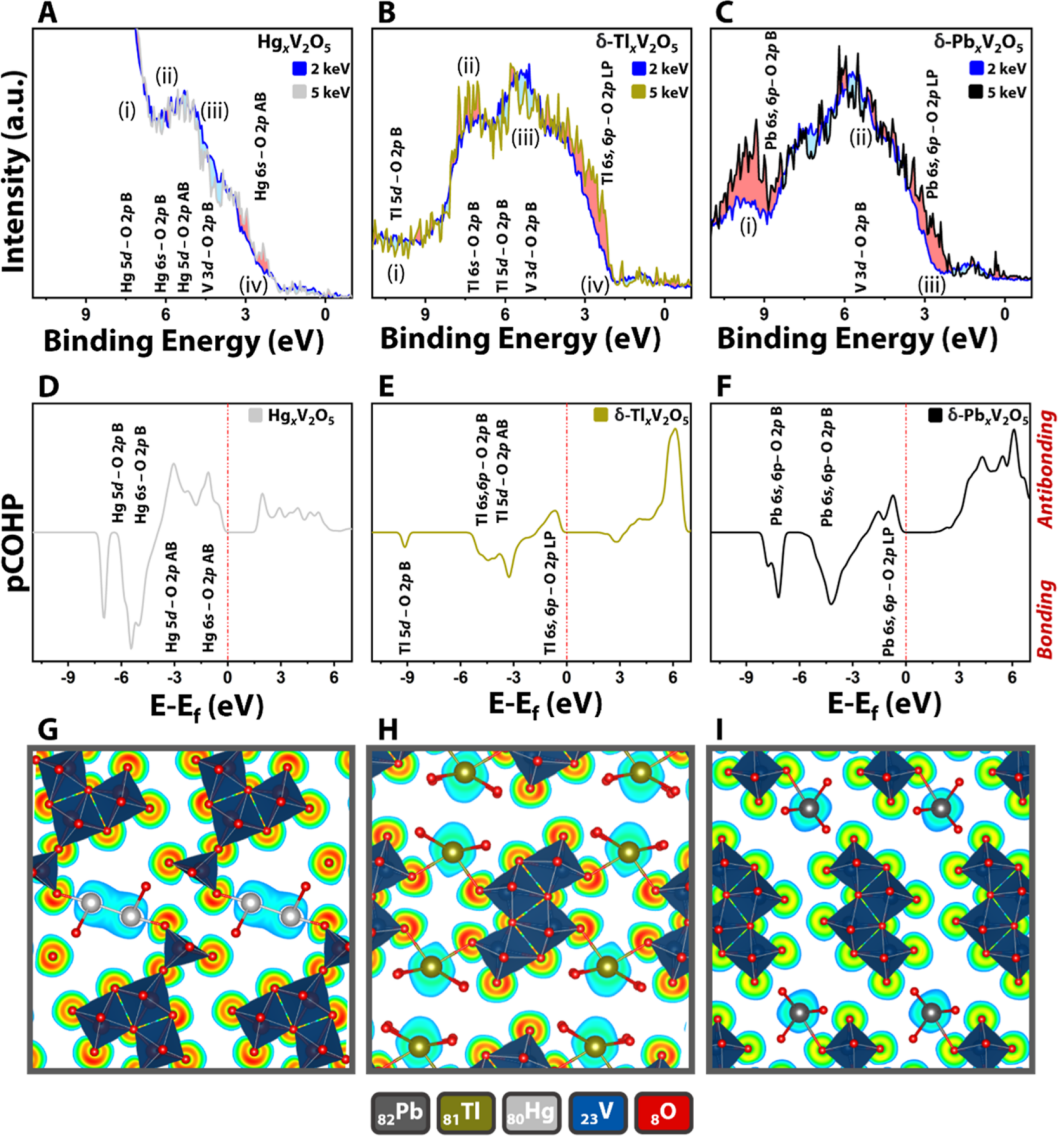
Electronic structure of M_0.5_V_2_O_5_ (M = Hg, Tl, and Pb). High-resolution HAXPES data was collected at incident photon energies of 2 and 5 keV for (A) Hg_0.5_V_2_O_5_, (B) δ-Tl_0.5_V_2_O_5_, and (C) δ-Pb_0.5_V_2_O_5_. Relative increases in the spectral weight of features as a function of incident photon energies are shaded in pink, whereas diminution is shaded in blue. COHP analyses of (D) Hg–O interactions in Hg_0.5_V_2_O_5_; (E) Tl–O interactions in δ-Tl_0.5_V_2_O_5_; and (F) Pb–O interactions in δ-Pb_*x*_V_2_O_5_. COHP bonding interactions between two species are negative on the vertical axis, whereas AB interactions are positive on the vertical axis. (G) ELF sliced along the (010) plane of Hg_0.5_V_2_O_5_. (H) ELF sliced along the (010) plane of δ-Tl_0.5_V_2_O_5_. (I) ELF sliced along the (010) plane of δ-Pb_0.5_V_2_O_5_.

To decipher the differences in the electronic structure of Hg_0.5_V_2_O_5_, δ-Tl_0.5_V_2_O_5_, and δ-Pb_0.5_V_2_O_5_, energy-variant valence band HAXPES measurements have been performed. Figure 2A–C compares the valence band HAXPES spectra collected at incident photon energies of 2.0 and 5.0 keV for Hg_0.5_V_2_O_5_, δ-Tl_0.5_V_2_O_5_, and δ-Pb_0.5_V_2_O_5_. The photoionization cross-sections of different orbitals decay sharply as a function of incident photon energy in photoemission spectroscopy; however, the exact dependences depend sensitively on the orbital angular momentum quantum number. As such, the HAXPES spectra acquired at higher incident photon energies (5 keV in this instance) more prominently feature orbital contributions from subshells with lower angular momentum values; this enables “spotlighting” of stereochemically active electron lone-pair states derived from filled 5s/6s-orbitals of p-block cations.^3,30−32^ Ternary vanadium oxides with a p-block cation have complex valence band spectra with contributions from p-block 5/6s states as well as V 3d and O 2p states. Energy-variant HAXPES thus serves as an excellent tool for probing the orbital characteristic of states at the valence band edge.

The HAXPES spectra plotted for Hg_0.5_V_2_O_5_ in Figure 2A shows four distinct features. In addition to the electronic states derived from Hg 5d orbitals, the valence band of Hg_0.5_V_2_O_5_ includes contributions from Hg 6s-, O 2p-, and V 3d-derived states^33,34^ as a result of the stabilization of distinctive Hg_2_^2+^ dimer motifs. The Hg_2_^2+^ dimer shows complex bonding with a considerable covalent characteristic and a substantial role for both relativistic effects and electron correlation (but relatively limited manifestation of spin–orbit coupling).^34,35^ First-principles electronic structure calculations have been performed for Hg_0.5_V_2_O_5_ as reported in the Methods section. Figure S3 plots the density of states. These plots provide insight into the relative energy positioning of the electronic states derived from various atomic orbitals. However, to better understand the hybridization of atomic orbitals in the valence band of Hg_0.5_V_2_O_5_ and to aid interpretation of HAXPES spectra, COHP analyses have further been performed.^36^Figure 2D plots pairwise bonding and antibonding interactions of Hg–O, which are used in concert with DOS calculations to assign the spectroscopic feature in HAXPES data. As evident from Figure 2D, both Hg 5d and 6s orbitals hybridize with O 2p states in the valence band. The hybridization of Hg 5d with O 2p leads to the formation of Hg 5d–O 2p bonding (B) and antibonding (AB) states. Similarly, the mixing of Hg 6s and O 2p leads to the formations of Hg 6s–O 2p B and AB states. The first and most intense feature observed deep in the valence band of Hg_0.5_V_2_O_5_ can be assigned to the electronic states deriving from Hg 5d states (Figure 2A). These Hg 5d states mix with the O 2p states, leading to Hg 5d–O 2p B states.

The mixing of shallow Hg 5d orbitals with O 2p has been previously observed in HgO.^37^ The second feature centered at 7 eV in the HAXPES spectra of Hg_0.5_V_2_O_5_ is a combination of Hg 5d–O 2p B and Hg 6s–O 2p B states. As a result of contributions from both Hg 5d–O 2p B and Hg 6s–O 2p B states, the former diminished and the latter enhanced with higher excitation energy, the intensity of the second feature in the valence band HAXPES spectrum of Hg_0.5_V_2_O_5_ does not change significantly as the photon energy is increased from 2 to 5 keV. The third feature in the HAXPES spectrum represents V 3d–O 2p B and Hg 5d–O 2p AB states. The relative intensity of this feature decreases slightly as a function of incident photon energy because of the d-orbital contributions to these states. The feature at the valence band maximum (VBM) is derived from the occupied Hg 6s–O 2p AB states. Since these states have a predominant s-orbital character, their relative intensity has somewhat greater spectral weight in the HAXPES spectra collected at 5 keV. Notably, the preponderant contribution to anion hybridization derives from Hg 5d–O 2p interactions; the Hg_2_^2+^ 6s^2^ lone pair predominantly mediates Hg–Hg interactions, is only weakly hybridized with O 2p states, and thus has minimal stereochemical activity.

The HAXPES spectra of δ-Tl_0.5_V_2_O_5_ in Figure 2B reveal four spectroscopic features. As compared to Hg 5d, Tl 5d states lie even deeper in the valence band and are less effective at hybridizing with O 2p states (as can be seen in the DOS plots in Figure S3). COHP analysis of δ-Tl_0.5_V_2_O_5_ suggests some hybridization of Tl 5d with O 2p, which results in the formation of Tl 5d–O 2p B states. The hybridization of Tl 5d with O 2p is substantially weaker in comparison to Hg 5d hybridization with O 2p as a result of poor orbital overlap resulting from the greater energetic differences between the atomic orbitals in the former interaction. A weak spectral feature originating from Tl 5d–O 2p B states is observed in the HAXPES spectrum of δ-Tl_0.5_V_2_O_5_ at ca. 10 eV (Figure 2B). The hybridization of Tl 6s^2^ states with O 2p leads to the formation of Tl 6s–O 2p B and Tl 6s–O 2p AB states. The second feature in the HAXPES spectrum of δ-Tl_0.5_V_2_O_5_ at ca. 7.5 eV can be assigned to Tl 6s–O 2p B states. The intensity of this feature increases with increasing incident photon energy owing to the substantial Tl 6s character. The third feature in the HAPXES spectra is derived from Tl 5d–O 2p AB and V 3d–O 2p AB states. As a result of the predominant d-orbital character of these states, the intensity of this feature is diminished with increasing photon energy. The Tl 6s–O 2p AB states are stabilized by hybridization with unoccupied Tl 6p states in the conduction band as per the revised lone-pair model,^38^ resulting in the formation of Tl 6s, 6p–O 2p hybrid states near the VBM. These states are canonical lone-pair states since they are derived from the overlap of Tl 6s, 6p with O 2p.^7^ As can be seen in Figure 2B, the intensity of these states markedly increases upon increasing the photon energy from 2 to 5 keV.

In δ-Pb_0.5_V_2_O_5_, the Pb 6s and 6p orbitals strongly hybridize with O 2p states as a result of the closely matched energy levels (manifested further as the short Pb(2)–O(6) bond and resulting superlattice ordering, as noted above). The hybridization of Pb 6s with O 2p leads to the formation of Pb 6s–O 2p B states deep in the valence band at ca. 10 eV, as observed in the HAXPES spectrum in Figure 2C. The Pb 6s–O 2p AB states are stabilized by further hybridization with Pb 6p, which yields Pb 6s, 6p–O 2p hybrid states at the VBM. The normalized intensity of both these features increases with increasing excitation energy owing to their significant Pb 6s character. Stereochemically active electron lone pairs of Pb have been previously observed in the HAXPES spectra of β-Pb_*x*_V_2_O_5_ and PbVO_3_Cl, wherein they underpin MITs and thermochromic behavior, respectively.^7,11^ The hybridization of V 3d with O 2p states leads to the formation of V 3d–O 2p B states in the valence band; however, these states show greater energy dispersion. The increased dispersion of these states arises from the different nominal vanadium valences in δ-Pb_0.5_V_2_O_5._

**Figure 3:**
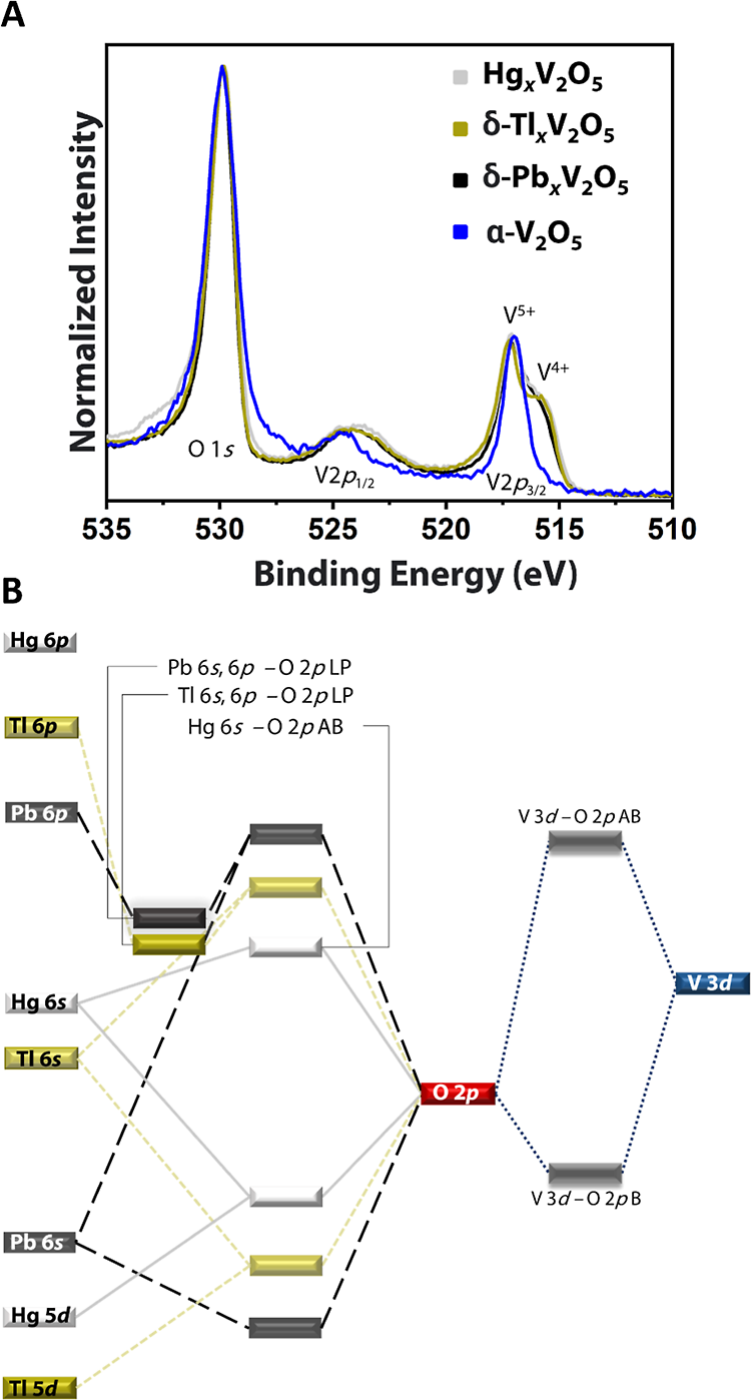
Molecular orbital perspective of stereochemical activity. (A) HAXPES spectra and O 1s and V 2p core levels collected at an incident photon energy of 2.0 keV for Hg_0.5_V_2_O_5_, δ-Tl_0.5_V_2_O_5_, δ-Pb_0.5_V_2_O_5_, and α-V_2_O_5._ The V2p_3/2_ feature shows the mixed valence of vanadium centers (nominally V^4+^ and V^5+^) in Hg_0.5_V_2_O_5_, δ-Tl_0.5_V_2_O_5_, and δ-Pb_0.5_V_2_O_5_. (B) Molecular orbital schematic sketching the hybridization between different atomic orbitals in Hg_0.5_V_2_O_5_, δ-Tl_0.5_V_2_O_5_, and δ-Pb_0.5_V_2_O_5_.

Figure 3A plots the V 2p and O 1s core-level HAXPES spectra collected at an excitation energy of 2 keV for α-V_2_O_5_, Hg_0.5_V_2_O_5_, δ-Tl_0.5_V_2_O_5_, and δ-Pb_0.5_V_2_O_5_. The V 2p_3/2_ feature at α-V_2_O_5_ shows a singular feature corresponding to the formally pentavalent vanadium centers illustrating the rich redox chemistry of vanadium that makes them excellent intercalation hosts. Intercalation of Hg, Tl, and Pb reduces some V centers from +5 to +4 in the V_2_O_5_ framework to maintain charge balance. For instance, δ-Pb_0.5_V_2_O_5_ can be written with formal charges as δ-(Pb^2+^)_0.5_(V^4+^)_1_(V^5+^)_1_O_5_. A clear shoulder corresponding to nominally tetravalent vanadium centers is observed for δ-Hg_0.5_V_2_O_5_, δ-Tl_0.5_V_2_O_5_, and δ-Pb_0.5_V_2_O_5_. To obtain an insight into electron localization and formal valence across the various V crystallographic sites, bond valence sum (BVS) calculations have been performed and are listed in Table 1.^39,40^ The BVS values across the different crystallographically inequivalent vanadium sites in Hg_0.5_V_2_O_5_ and δ-Tl_0.5_V_2_O_5_ show a relatively narrow spread. However, a much greater separation in electron localization is observed across the six crystallographically inequivalent centers of δ-Pb_0.5_V_2_O_5_ with charges varying from 4.39 to 5.0 (Table 1). Indeed, this suggests an electron localization motif, which is furthermore consistent with the short Pb–O bond and supercell ordering of Pb cations observed in Figure 1H.

**Table 1:** Interatomic Distances (Å) and BVS for M_*x*_V_2_O_5_ (M = Hg, Tl, and Pb)^a^

bond distance (Å)		bond distance (Å)		bond distance (Å)		bond distance (Å)	
Hg_0.5_V_2_O_5_		δ-Tl_0.5_V_2_O_5_		δ-Pb_0.5_V_2_O_5_			
V1–O1	1.600	V1–O1 (×2)	1.913	V1–O1	1.592	V4–O3	2.536
V1–O2	1.940	V1–O2	1.602	V1–O2 (×2)	1.929	V4–O8	1.712
V1–O4	1.926	V1–O3	1.836	V1–O3	1.957	V4–O9	2.027
V1–O5	2.518	V1–O4	2.541	V1–O8	1.964	V4–O10	1.584
V1–O10 (×2)	1.895	V1–O5	1.975	V1–O8	2.251	V4–O11 (×2)	1.907
**BVS**	**4.600**	**BVS**	**4.640**	**BVS**	**4.556**	**BVS**	**5.016**
V2–O3	1.601	V2–O1	2.030	V3–O2 (×2)	1.983	V6–O5	2.301
V2–O4	1.944	V2–O3	1.818	V3–O5	1.926	V6–O7	1.908
V2–O6	1.986	V2–O3	2.345	V3–O6	1.603	V6–O11 (×2)	1.940
V2–O7	2.383	V2–O4	1.617	V3–O7	1.930	V6–O14	1.952
V2–O7 (×2)	1.891	V2–O5 (×2)	1.894	V3–O7	2.417	V6–O15	1.639
**BVS**	**4.564**	**BVS**	**4.701**	**BVS**	**4.707**	**BVS**	**4.389**
V3–O4	2.021			V2–O2	1.983	V5–O9	1.885
V3–O5	1.591			V2–O3	1.926	V5–O11	2.037
V3–O6 (×2)	1.898			V2–O4	1.603	V5–O12	1.847
V3–O7	2.277			V2–O5	1.930	V5–O12	2.304
V1–O8	1.995			V2–O11	2.417	V5–O13	2.628
**BVS**	**4.511**			V2–O12	1.810	V5–O14	1.906
V4–O2 (×2)	1.913			**BVS**	**4.683**	**BVS**	**4.598**
V4–O8	1.666						
V4–O9	1.660						
V4–O10	2.165						
**BVS**	**4.542**						

a BV = e^(*r*_0_–*r*)/*b*^ with the following parameters: *b* = 0.37 and *r*_0_(V–O) = 1.784 Å

Electron localization function (ELF) maps have furthermore been calculated for Hg_0.5_V_2_O_5_, δ-Tl_0.5_V_2_O_5_, and δ-Pb_0.5_V_2_O_5_. The ELF is symmetrically disposed around Hg centers in the case of Hg_0.5_V_2_O_5_, which suggests that the electron lone pair on Hg centers is essentially stereochemically inactive. This observation is fully consistent with the HAXPES and COHP data in Figure 2. However, in the case of Tl centers in δ-Tl_0.5_V_2_O_5_, the ELF maps in Figure 2G–I show greater asymmetry. As observed in HAXPES data, the stabilization of Tl 6s–O 2p AB states mediated by hybridization with Tl 6p as per a second-order Jahn–Teller distortion yields AB Tl 6s, 6p–O 2p lone-pair states at the VBM.^41^ Being occupied AB states, they give rise to electron localization in AB orbitals around Tl, which is reflected as depletion of electron density from Tl–O bonds. In the ELF map for δ-Pb_0.5_V_2_O_5_, once again, considerable electron depletion from Pb–O bonds is observed, and the ELF is strongly distorted around the Pb centers, denoting strong stereochemical activity of Pb 6s^2^ states, as also observed in HAXPES plots and COHP data.

Figure 3B presents a molecular orbital perspective of the electronic structure of all the three compounds to explain the increasing stereochemical activity from Hg_0.5_V_2_O_5_ to δ-Pb_0.5_V_2_O_5_. In Hg_0.5_V_2_O_5_, primarily, the Hg 5d and to a lesser extent 6s orbitals hybridize with O 2p (an effective bond order of 0 is inferred from COHP analyses), whereas the Hg 6p states are relatively higher in energy. The Hg 6s states are furthermore engaged in the stabilization of the [Hg–Hg]^2+^ dimer (not shown),^33,35^ and thus, the density of Hg 6s–O 2p states at the VBM is low. In δ-Tl_0.5_V_2_O_5_, Tl 5d, 6s, and 6p states hybridize with O 2p, and the extent of hybridization of Tl 6s states is considerably greater, resulting in more of a stereochemically active lone pair. In δ-Pb_0.5_V_2_O_5_, the 6s and 6p states are strongly hybridized with O 2p, yielding mid-gap states with canonical stereochemically active lone-pair character at the VBM.^7^ The V 3d orbitals hybridize with O 2p orbitals in all the three ternary vanadium oxides yielding mixed valent character. The conduction band edge is dominated by unoccupied V 3d states (Figure S3). In summary, the electronic structure measurements reveal that 6s^2^-derived states from intercalated Hg_2_^2+^ centers are stereochemically inactive in Hg_0.5_V_2_O_5_; however, the stereochemically active electron pairs are present for both Tl and Pb centers in δ-Tl_0.5_V_2_O_5_ and δ-Pb_0.5_V_2_O_5_, respectively, with the latter showing a pronounced lattice distortion that engenders a distinctive supercell ordering motif (Figure 1H).

### Magnetic Ordering

**Figure 4:**
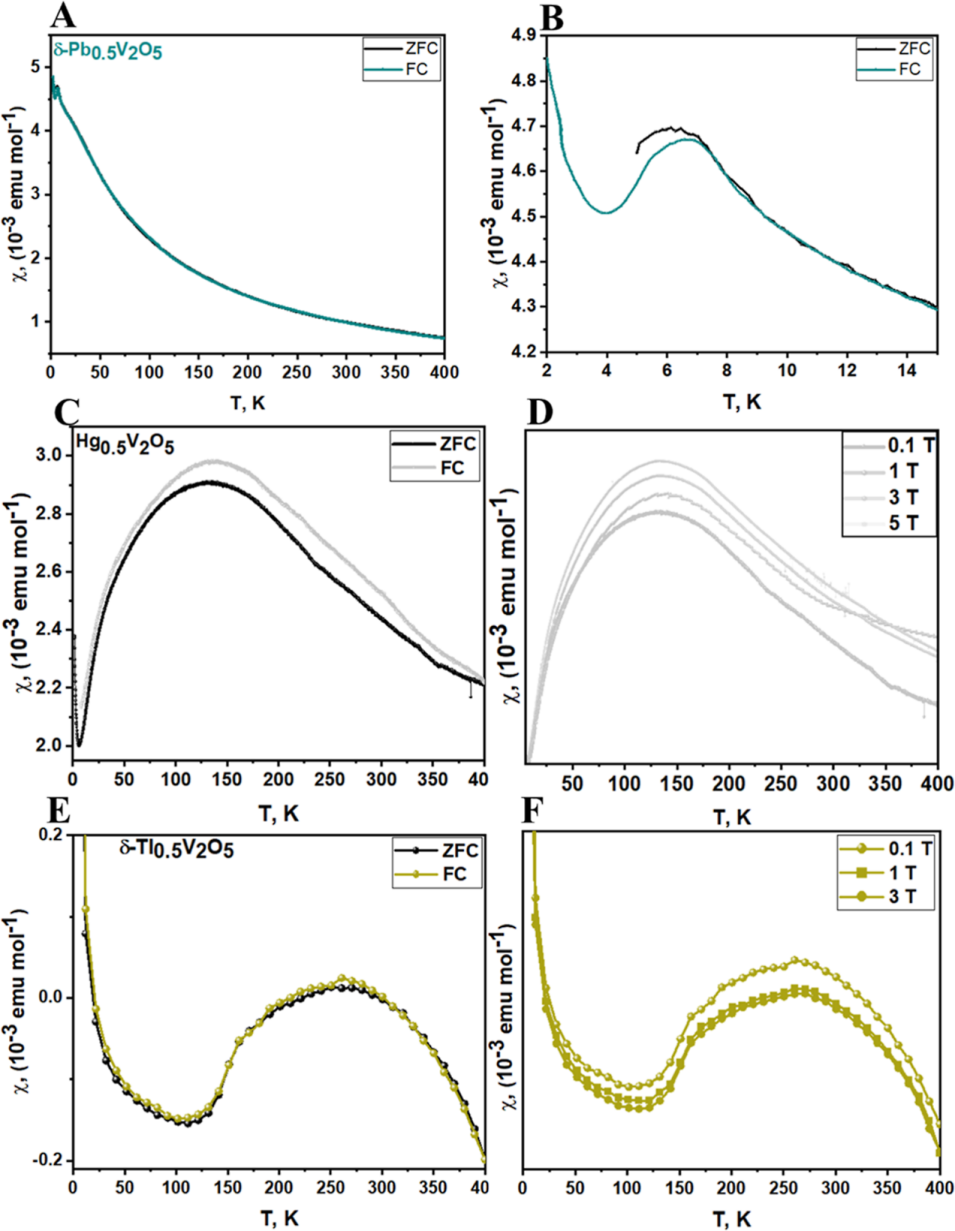
Magnetic behavior of M_0.5_V_2_O_5_ (M = Pb, Tl, and Hg). (A) ZFC and FC magnetic susceptibility of δ-Pb_0.5_V_2_O_5_ at μ_0_(H) = 0.1 T. (B) Low-temperature dependence of the magnetic susceptibility of δ-Pb_0.5_V_2_O_5_ at μ_0_(H) = 0.1 T. (C) ZFC and FC magnetic susceptibility of Hg_0.5_V_2_O_5_ under an applied field of 0.1 T. (D) Temperature dependence of the magnetic susceptibility of Hg_0.5_V_2_O_5_ at different magnetic fields. (E) ZFC and FC magnetic susceptibility of δ-Tl_0.5_V_2_O_5_ under an applied field of 0.1 T. (F) Temperature dependence of the magnetic susceptibility of δ-Tl_0.5_V_2_O_5_ at different magnetic fields.

**Figure 5:**
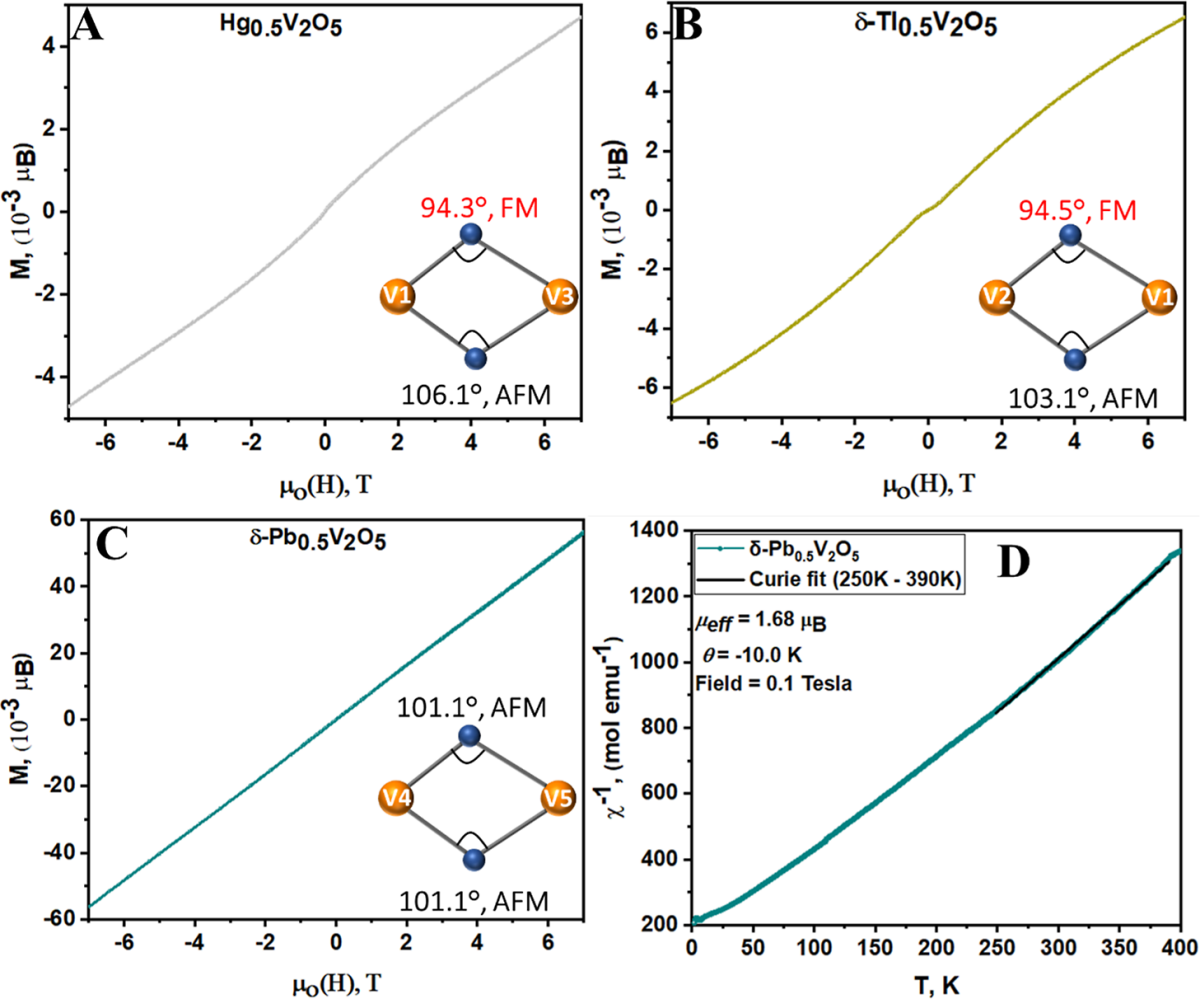
Probing the magnetic behavior of M_0.5_V_2_O_5_ (M = Pb, Tl, and Hg). Magnetization vs magnetic field curve plotted for (A) Hg_0.5_V_2_O_5_, (B) δ-Tl_0.5_V_2_O_5_, and (C) δ-Pb_0.5_V_2_O_5_ at a temperature of 5 K. (D) Inverse magnetic susceptibility of δ-Pb_0.5_V_2_O_5_ in the field-cooled state. The inverse magnetic susceptibility has been fit to the Curie–Weiss law from 250 to 390 K; the relevant fit parameters obtained are presented within the panel. No hysteresis is observed at 5 K.

The temperature dependence of the magnetic susceptibility, χ(T), of δ-Pb_0.5_V_2_O_5_, Hg_0.5_V_2_O_5_, and δ-Tl_0.5_V_2_O_5_ has been measured under zero-field cooled (ZFC)–FC (field cooled) conditions in the temperature range of 2–400 K under an external field of 0.1 T as shown in Figure 4. The ZFC and FC susceptibilities increase with decreasing temperature. A relatively broad maximum is observed around 7 K for δ-Pb_0.5_V_2_O_5_, 160 K for Hg_0.5_V_2_O_5_, and 260 K for δ-Tl_0.5_V_2_O_5_, suggesting the presence of AFM ordering at low temperatures in these systems. The ZFC–FC susceptibility curve shows a divergence at *T* < *T*_N_ for δ-Pb_0.5_V_2_O_5_ (Figure 4B) as well as at *T* > *T*_N_ for Hg_0.5_V_2_O_5_ (Figure 4C) and becomes minimal at *T* < *T*_N_. This divergence of Hg_0.5_V_2_O_5_ between ZFC and FC disappears as the field strength increases (Figure S4). Additionally, the *T*_N_ of Hg_0.5_V_2_O_5_ changes slightly with applied magnetic fields 0.1 T < μ_0_(H) < 5 T (Figure 4D). As in the case of Hg_0.5_V_2_O_5_, the *T*_N_ value of δ-Tl_0.5_V_2_O_5_ increases slightly with applied magnetic fields of 1 and 3 T (Figure 4F), with a weak hysteretic behavior below *T*_N_, which disappears with increasing magnetic field (Figure S5). Further evidence of the AFM ordering of V 3d^1^ centers is observed in Figure 5A–C, where the magnetization is plotted against the magnetic field and exhibits a negative curvature. At 5 K, the compounds show a linear increase in magnetization with increasing applied field and negligible hysteresis; the saturation magnetization values are inferred as 0.055, 0.0045, and 0.0063 μ_B_/V for δ-Pb_0.5_V_2_O_5_, Hg_0.5_V_2_O_5_, and δ-Tl_0.5_V_2_O_5_, respectively.

The upturn of susceptibilities seen at 4, 5, and 110 K in δ-Pb_0.5_V_2_O_5_, Hg_0.5_V_2_O_5_, and δ-Tl_0.5_V_2_O_5_, respectively, is most likely a result of isolated spins. Alternatively, it might be a result of ferromagnetic exchange coupling arising from a canted-spin arrangement of V^*n*+^ centers. The temperature at which this upturn occurs is decreased and eventually disappears with increasing applied field for Hg_0.5_V_2_O_5_ (Figure S4) but remains unchanged for δ-Tl_0.5_V_2_O_5_ even up to 3 T (Figure S5). All the three compounds under consideration manifest paramagnetic behavior at *T* > *T*_N_, which follows a Curie–Weiss dependence for paramagnetic spins (Figures 5D and S6A,B)
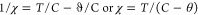
1where ϑ and *C* are the Curie–Weiss temperature and Curie constant, respectively. The effective magnetic moments μ_eff_ of the V^*n*+^ centers estimated from the resulting Curie constant are 1.68, 1.75, and 1.74 μ_B_ for δ-Pb_0.5_V_2_O_5_, Hg_0.5_V_2_O_5_, and δ-Tl_0.5_V_2_O_5_, respectively. These values are close to the ideal *g* = 1.73 μ_B_ value expected for a free *S* = 1/2 moment. The Curie–Weiss temperature is negative (δ-Pb_0.5_V_2_O_5_ = −10 K, Hg_0.5_V_2_O_5_ = −172 K, and δ-Tl_0.5_V_2_O_5_ = −280 K), indicating a dominant AFM-exchange interaction between the V^*n*+^ centers.

**Figure 6:**
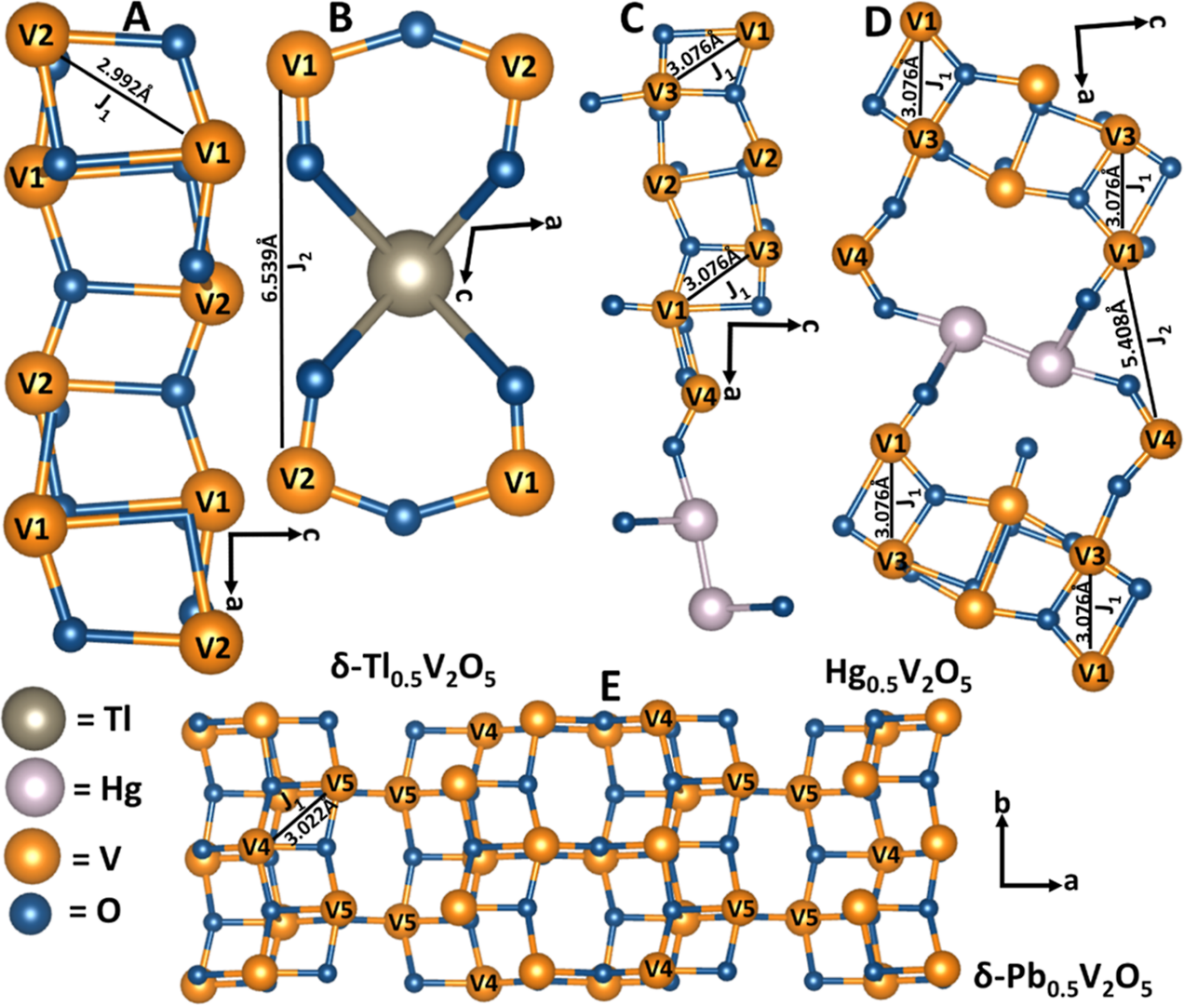
Possible magnetic superexchange interaction pathways in 2D M_*x*_V_2_O_5_. (A) Nearest-neighbor (*J*_1_) and (B) next-nearest neighbor (*J*_2_) magnetic exchange interactions in δ-Tl_0.5_V_2_O_5_. Magnetic exchange constants of (C) nearest and (D) next-nearest-neighbor interactions as denoted by *J*_1_ and *J*_2_, respectively, in Hg_0.5_V_2_O_5_. (E) Nearest-neighbor (*J*_1_) magnetic exchange interactions in δ-Pb_0.5_V_2_O_5_. *J*_2_ includes rather extended (Hg_0.5_V_2_O_5_ = 5.408(6) Å and δ-Tl_0.5_V_2_O_5_ = 6.539(7) Å) magnetic exchange pathways, suggesting a much weaker magnetic interaction as compared to those expected for *J*_1_ (δ-Pb_0.5_V_2_O_5_ = 3.022(4) Å, Hg_0.5_V_2_O_5_ = 3.076(7) Å, and δ-Tl_0.5_V_2_O_5_ = 2.992(6) Å). Considering the V–V bond distances in Table S9, all the bond distances are slightly longer than the cation–cation critical distance (*R*_c_ = 2.94 Å). As a result, a direct (V^*n*+^–V^*n*+^) d-orbital overlap is unlikely. Alternative *J*_2_ (V^*n*+^–O···M–M···O–V^*n*+^) interaction includes long magnetic exchange pathways, suggesting a much weaker magnetic interaction as compared to those expected for *J*_1_ (δ-Pb_0.5_V_2_O_5_ = 3.022(4) Å, Hg_0.5_V_2_O_5_ = 3.076(7) Å, and δ-Tl_0.5_V_2_O_5_ = 2.992(6) Å).

The variation in the AFM transition temperature across the three compounds derives from their distinctive exchange coupling mechanisms, which in turn are governed by the different bond distances and angles enforced by the intercalated p-block cation. Insertion of cations between the [V_4_O_10_] layers and the resulting local lattice distortions induced as a result of stereochemical (in)activity of 6s^2^ lone pairs strongly modifies V–O bonds and the pattern of electron localization across the framework. As such, the intercalated p-block cations affect substantial variations in V–V separations along the chains of vanadium-centered polyhedra in the infinite [V_4_O_10_]-condensed double layers, resulting in distinctive magnetic interaction pathways. Based on the crystal structure of the three compounds under consideration, three possible exchange pathways are sketched in Figure 6: V^*n*+^–V^*n*+^, V^*n*+^–O–V^*n*+^(*J*_1_), and V^*n*+^–O···M–M···O–V^*n*+^(*J*_2_). Considering the V–V bond distances inferred from Table S9, superexchange coupling between the V 3d^1^ centers through oxygen 2p orbitals is the most probable (V^*n*+^–O–V^*n*+^) as sketched in the insets of Figure 5A–C.

In Hg_0.5_V_2_O_5_ and δ-Tl_0.5_V_2_O_5_, the BVS values of the different crystallographically inequivalent V sites are similar (Table 1). As such, superexchange interactions are mediated across the *ab* planes of the [V_4_O_10_] slabs. The higher transition temperature of δ-Tl_0.5_V_2_O_5_ as compared to Hg_0.5_V_2_O_5_ can be ascribed to the accessibility of an intra-chain (V1–O1–V2 = 2.992(6) Å) exchange pathway (Figure 6A), which runs across the *ab* planes of the [V_4_O_10_] slabs. However, in Hg_0.5_V_2_O_5_, the Hg_2_^2+^ dimers disrupt these chains, necessitating a nonlinear/discontinuous exchange pathway across V1–V3 (Figure 6C,D). The V1–O4–V3 distance is considerably longer at 3.076(7) Å, which thus results in a lower transition temperature. Conversely, in δ-Pb_0.5_V_2_O_5_ (Figure 6E), repulsions between stereochemically active electron lone pairs enforce a superstructure with a checkerboard cation ordering motif, as shown in Figure 1H. This in turn results in periodic electron localization along the [V_4_O_10_] layers and large variations in vanadium valence (Table 1). This charge ordering diminishes both intra- and inter-chain-exchange couplings, leading to a lower *T*_N_. It is noteworthy to mention that all the bond angles involved in the superexchange interactions are >90° (V4–O9–V5 = 101.1(5)° for δ-Pb_0.5_V_2_O_5_, V1–O1–V2 = 103.1(4)° for δ-Tl_0.5_V_2_O_5_, and V1–O4–V3 = 106.1(9)° for Hg_0.5_V_2_O_5_ as shown in Figure S7 and the insets of Figure 5A–C), which further confirms the dominant AFM behavior of these systems.^42^ Based on these structural considerations, the weak FM (S-character) observed in the magnetization curve of Hg_0.5_V_2_O_5_ (Figure 5A) and δ-Tl_0.5_V_2_O_5_ (Figure 5B) can be attributed to the competing bond angles of V1–O4–V2 = 94.5(5)° for δ-Tl_0.5_V_2_O_5_ and V1–O5–V3 = 94.3(9)° for Hg_0.5_V_2_O_5_, as shown in the insets of Figure 5A,B. In contrast, this S-characteristic is absent in δ-Pb_0.5_V_2_O_5_ (Figure 5C), where the corresponding V4–O11–V5 bond angle is 101.1(3)° as shown in the inset of Figure 5C.

## Conclusions

The double-layered δ, λ, and ρ polymorphs of V_2_O_5_ comprise infinite slabs of condensed [V_4_O_10_] bilayers that differ only in the stacking sequence of the double layers.^43^ The galleries between the layers can accommodate a broad range of s-, p-, and d-block cations across a range of interstitial sites including numerous examples of solvated cations. Here, we use these quasi-2D layered frameworks as testbeds for systematically modulating the electronic structure, Fermi surfaces, and magnetic ordering by positioning p-block cations with 6s^2^ electron lone pairs in interstitial sites between the double layers. In canonical lone pair compounds such as BiVO_4_, CsPbBr_3_, and GeTe wherein the p-block cation is an intrinsic structural element, systematic compositional modifications can be challenging to explore without transforming to an entirely different structure type. In contrast, in the intercalation strategy pursued here, a broad range of p-block pillaring cations can be positioned in interstitial sites while preserving the overall structural connectivity of the host framework. This allows for systematic modulation of lone-pair–anion hybridization. Comparing 6s^2^ cations, Hg_2_^2+^, Tl^+^, and Pb^2+^ positioned in the interlayer sites of the δ-V_2_O_5_ polymorph, we note a smooth gradation from inert to stereochemically active characteristic as determined by energy-variant HAXPES, COHP analyses of pairwise bonding and antibonding interactions, and mapping of ELFs around the inserted cations.

The three period VI cations show distinctive bonding motifs and local coordination environments, which are determined by the strength of anion hybridization. The Hg 6s electrons mediate the Hg_2_^2+^ dimer formation in a Heitler–London picture; however, filled 6s and unfilled 6p Hg orbitals are mismatched in energy for effective hybridization with O 2p states. As such, the electron density remains symmetrically disposed around interstitially positioned Hg ions, and the Hg 6s^2^ electrons are best considered as inert pairs. In contrast, in δ-Tl_*x*_V_2_O_5_, strong Tl 6s hybridization is observed with O 2p states, mediated by second-order Jahn–Teller mixing with Tl 6p states, as predicted by the revised lone-pair model;^38^ the stereochemical activity of the Tl 6s^2^ lone pairs induce substantial local structural distortions. Consistent with this trend, δ-Pb_0.5_V_2_O_5_ has the most energetically favored positioning of Pb 6s and 6p states for hybridization with O 2p states and shows strong stereochemical activity. Electron lone-pair repulsions are sufficiently pronounced in this structure to enforce superlattice ordering of Pb-cations, as inferred from powder XRD studies. The lone-pair-derived local structural distortions strongly modify V–O bonding and electron localization along the [V_4_O_10_] slabs. HAXPES data also reveal a progressive decrease in the amount of O 2p characteristic at the VB edges in going from group 12 to group 14 cations.

Competing ferromagnetic-antiferromagnetic superexchange interactions are observed with AFM ordering mediated by superexchange through O 2p states being the primary coupling mechanism between spins localized on the [V_4_O_10_] framework. Magnetic ordering temperatures of 160, 260, and 7 K are observed for Hg_0.5_V_2_O_5_, δ-Tl_0.5_V_2_O_5_, and δ-Pb_0.5_V_2_O_5_, respectively. A strong coupling chain between distorted VO_6_ octahedra is sundered by Hg_2_^2+^ dimers, thereby decreasing the ordering temperature. Superstructure ordering of cations and the resulting charge ordering greatly diminishes coupling pathways in δ-Pb_0.5_V_2_O_5_, resulting in an anomalously low ordering temperature. In summary, our findings open new avenues to use stereochemically active cations strategically positioned in interstitial sites of insertion hosts to tune lattice anharmonicity, electronic structure, and magnetic ordering. In addition to affording a filled mid-gap state at the VBM that can be potentially used for redox catalysis, the strategy demonstrated here illustrates the promise of using stereochemically active electrons to finely modulate superexchange as well as electron correlation with potential implications for driving discontinuous electronic and magnetic transitions needed for information processing.

## Experimental Section

### Synthesis of δ-M_*x*_V_2_O_5_

Layered δ-M_*x*_V_2_O_5_ bronzes (M = Tl and Pb) were synthesized by adapting previously reported methods.^29,44^ Briefly, the materials were prepared by the reaction between bulk as-obtained orthorhombic V_2_O_5_ and the desired metal acetate salt as follows
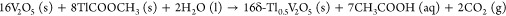

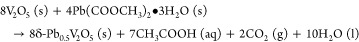


To prepare δ-Tl_*x*_V_2_O_5_, TlCOOCH_3_ (126.0 mg, Sigma-Aldrich) and V_2_O_5_ (174.0 mg, Beantown Chemical, 99.5%) were dispersed in a 16 mL aqueous solution of 1.1 M acetic acid in a 1:2 molar ratio. To prepare δ-Pb_*x*_V_2_O_5_, Pb(COOCH_3_)_2_·3H_2_O (183.0 mg, Sigma-Aldrich) and V_2_O_5_ (117.0 mg, Beantown Chemical, 99.5%) in a 3:4 molar ratio (excess Pb to promote the formation of the layered phase as compared to quasi-1D β-Pb_0.33_V_2_O_5_) were dispersed in a 16 mL aqueous solution of 1.1 M acetic acid. The dispersions were subsequently added to a 23 mL capacity polytetrafluorethylene-lined stainless steel autoclaves (Parr). In both cases, the hydrothermal reactions were allowed to proceed for 72 h at 250 °C. Following the reaction, the autoclaves were allowed to cool to room temperature. The contents of the PFTE liners were filtered by vacuum filtration, and the materials were washed with copious amounts of deionized water and 2-propanol. Emerald-green δ-Tl_*x*_V_2_O_5_ and shimmering black/purple δ-Pb_*x*_V_2_O_5_ powders were recovered by filtration.

Single crystals of δ-Pb_0.5_V_2_O_5_ and δ-Tl_0.5_V_2_O_5_ were obtained by sealing the prepared powder samples under vacuum in a fused silica ampoule, melting at 800 °C in a programmable furnace (Thermo Scientific, Lindberg Blue M with UT150 controller), and cooling through the melting point at a rate of 2 °C/h. δ-Pb_0.5_V_2_O_5_ and δ-Tl_0.5_V_2_O_5_ formed in the habit of long, thin plates, with lustrous black δ-Pb_0.5_V_2_O_5_ crystals and translucent green δ-Tl_0.5_V_2_O_5_ crystals.

Powder samples of Hg_0.5_V_2_O_5_ were prepared by solid-state synthesis by adapting a previously reported synthesis.^45^ A mixture of elemental Hg (421.3 mg, Sigma-Aldrich, 99% purity) and V_2_O_5_ (578.7 mg, Sigma-Aldrich, 99% purity) in a 1:2 molar ratio (total weight ca. 1.0 g) was ball-milled in a Spex Certiprep ball-mill for 1 h using polystyrene beads as the milling media. In light of its volatility, a 32% molar excess of Hg was added to the mixture. The resulting mixture was placed within a fused silica ampoule and sealed under vacuum. The ampoule was heated to 550 °C for 72 h and then cooled to room temperature. The powder products were visibly black at room temperature; these stoichiometries minimized the presence of unreacted precursors.

### Caution

Due caution needed to be exercised when working with Tl and Hg compounds! The thallium acetate precursor salt, Hg, and final products need to be handled using appropriate personal protective equipment and with due engineered controls given the volatility of mercury. The powders should not be breathed-in or allowed to contact skin. The supernatant from the hydrothermal reaction of δ-Tl_0.5_V_2_O_5_ will also have some residual solubilized Tl species. The hydrothermal vessel should be opened in a vented fume hood, and the residual waste should be carefully labeled.

### Structure Elucidation

Powder XRD data were collected using a using a Bruker D8-Focus diffractometer (Cu Kα: λ = 1.5418 Å, 40 kV voltage, and 25 mA current). Data were collected over the angle range 10°≤ 2θ ≤ 90° with a step width of 0.008° and a step rate of 0.0557°/s at room temperature. Rietveld refinements were performed using the EXPGUI user interface of GSAS.^46^ Atomic positions, profile terms, lattice parameters, and inserted metal occupancies were refined from the laboratory XRD data using isotropic thermal parameters. All crystal structure renditions were prepared using the Vesta III software suite (JP-Minerals).^47^

Single-crystal diffraction data were collected on a BRUKER Quest X-ray diffractometer utilizing the APEX3 software suite; X-ray radiation was generated from a Mo-I μs X-ray tube (*K*_α_ = 0.71073 Å). All crystals were placed in a cold N_2_ stream maintained at 110 K. Following unit cell determination, extended data collection was performed using omega and phi scans. Data reduction, integration of frames, merging, and scaling were performed with the program APEX3, and absorption correction was performed utilizing the program SADABS.^48,49^ Structures were solved using intrinsic phasing. Least-squares refinement for all structures was carried out on *F*^2^. Structural refinement, the calculation of derived results, and the generation of electron density maps were performed using the SHELXTL package of computer programs, ShelXle, and Olex2.^50−52^ A Crystallographic Information File for δ-Tl_0.5_V_2_O_5_ has been deposited in the Cambridge Structural Database and is available for access with deposition number 2170921.

### Electron Microscopy

SEM images were obtained using a JEOL JSM-7500F FE-SEM equipped with an Oxford energy-dispersive X-ray spectrometer (EDS) for elemental characterization. SEM images were collected at an accelerating voltage of 5 kV; EDS spectra were collected at an accelerating voltage of 20 kV. Low-magnification TEM images were collected using a JEOL JEM-2010 electron microscope at an operating voltage of 200 kV. Prior to imaging, powder materials were affixed to a conductive carbon tape (SEM) or dispersed in 2-propanol and drop-cast onto a copper grid (TEM).

### HAXPES Measurements

HAXPES measurements were performed at the National Institute of Standards and Technology (NIST) beamline SST-2 of the National Synchrotron Light Source II of Brookhaven National Laboratory. Measurements were performed at approximately 2 keV photon energy with a pass energy of 200 eV and a step size of 0.85 eV with the analyzer axis oriented parallel with the photoelectron polarization vector. HAXPES data at 5 keV was collected with a 500 eV energy filter. The higher excitation energy of HAXPES circumvents deleterious charging issues that are ubiquitous in ultraviolet and soft X-ray photoelectron spectroscopy.^53^ Photon energy selection was accomplished using a double Si (111) crystal monochromator. No evidence of charging was observed during our measurements. The beam energy was aligned to the Fermi level of a silver foil before measurements.

### Magnetic Measurements

Magnetic measurements of Hg_0.5_V_2_O_5_, δ-Pb_0.5_V_2_O_5_, and δ-Tl_0.5_V_2_O_5_ powders were carried out on a Quantum Design Magnetic Property Measurement System using the Quantum Design superconducting quantum interference device magnetometer option. Both ZFC and FC measurements have been performed in the temperature range of 2–700 K with an applied field of up to 0.01 T. Field-dependent magnetization measurements were performed at 5 K and above room temperature.

### Computational Methods

Electronic structure calculations were performed using density functional theory as implemented in the Vienna Ab Initio Simulation Package (VASP).^54−56^ Initial atomic positions for Hg_0.5_V_2_O_5_, δ-Tl_0.5_V_2_O_5_, and δ-Pb_0.5_V_2_O_5_ were obtained from structure solutions derived from the XRD data. The projected augmented wave formalism was used to model electron–ion interactions.^57^ A kinetic energy cutoff of 520 eV was used for plane-wave basis restriction. Electronic exchange and correlation effects were included using the generalized gradient approximation based on the Perdew–Burke–Ernzerhof functional (GGA-PBE).^58^ A Hubbard correction of *U* = 3.25 eV was used to account for strong electron correlation in the V 3d electrons as benchmarked in a previous study.^59^ A Monkhorst–Pack reciprocal grid of 2 × 4 × 4 points was used for the relaxation of 1 × 2 × 1 supercell structures. The structures were relaxed when each Cartesian force component was less than 0.01 eV/Å unless otherwise noted. ELF plots were produced by the VASP output in Vesta.^60^ COHP analyses were performed using the software package Local Orbital Suite Toward Electronic-Structure Reconstruction (LOBSTER).^36,61,62^ LOBSTER-recommended basis functions were for the projection calculations accounting for 2s and 2p orbitals of oxygen, 3d and 4s orbitals of vanadium, 5d and 6s orbitals of Hg, and 5d, 6s, and 6p orbitals of Tl and Pb. The absolute charge spilling is <3.56% in all cases.

## Supporting Information Available

Refinement statistics and structural details; bond distances and bond angles; selected V–V bond distances; electron microscopy characterization (SEM and EDS); density of states plots; additional magnetic susceptibility data; inverse magnetic susceptibility plots; and depictions of principal superexchange interaction pathways (PDF)
