# Supplementary material for: Toggling Stereochemical Activity through Interstitial Positioning of Cations between 2D V2O5 Double Layers
Source: Chem Mater. 2023 Aug 31;35(17):7175–88. doi: 10.1021/acs.chemmater.3c01463 (PMC10862490; doi:10.1021/acs.chemmater.3c01463)
Supplement: Supplementary file 1 — cm3c01463_si_001.pdf [file cm3c01463_si_001.pdf]

Toggling Stereochemical Activity through Interstitial Positioning of Cations between 2D V_2O_5 Double Layers

George Agbeworvi,[1,†] Wasif Zaheer,[1,†] Joseph V. Handy,[1] Justin L. Andrews,[1] Saul Perez-Beltran,[2] Cherno Jaye,[3] Conan Weiland,[3] Daniel A. Fischer,[3] Perla B. Balbuena, and Sarbajit Banerjee[1,*]

1. Department of Chemistry and Department of Material Science and Engineering, Texas A&M University, College Station, TX, 77843, USA;

2. Department of Chemical Engineering, Texas A&M University, College Station, TX, 77843, USA;

3. Material Measurement Laboratory, National Institute of Standards and Technology, Gaithersburg, MD, 20899, USA

† Equal contribution

* Corresponding author

Corresponding Author's Email Address: banerjee@chem.tamu.edu

Table S1. Refinement statistics and structural details for the structure of $Hg_xV_2O_5$ shown in Fig. 1B, which has been obtained from Rietveld refinement of the powder XRD data shown in Fig. 1A (monoclinic, *C 2/m*).

a = 22.3550(1) Å, b = 3.63120(2) Å, c = 9.61130(4) Å, β = 91.708(5)°, V = 780.55(6) Å^3 χ^2 = 8.74, Rp = 6.29%, wRp = 9.36%					
**Atom**	***x***	***y***	***z***	**Occupancy**	**Uiso**
**Hg(1)**	0.4816(5)	0.00(0)	0.3754(11)	1.00(0)	0.022(5)
**V(1)**	0.1199(19)	0.00(0)	0.1988(4)	1.00(0)	0.011(10)
**V(2)**	0.1881(19)	0.00(0)	0.5346(4)	1.00(0)	0.011(10)
**V(3)**	0.2571(19)	0.00(0)	0.2304(5)	1.00(0)	0.011(10)
**V(4)**	0.3985(19)	0.00(0)	0.0582(4)	1.00(0)	0.009(10)
**O(1)**	0.0602(8)	0.00(0)	0.2860(2)	1.00(0)	0.022(5)
**O(2)**	0.0962(8)	0.00(0)	0.0030(2)	1.00(0)	0.020(4)
**O(3)**	0.1191(8)	0.00(0)	0.5744(19)	1.00(0)	0.018(4)
**O(4)**	0.1795(7)	0.00(0)	0.3327(19)	1.00(0)	0.013(4)
**O(5)**	0.2201(8)	0.00(0)	0.0865(19)	1.00(0)	0.015(4)
**O(6)**	0.2292(7)	0.00(0)	0.7206(18)	1.00(0)	0.012(4)
**O(7)**	0.2884(7)	0.00(0)	0.4580(19)	1.00(0)	0.013(4)
**O(8)**	0.3406(8)	0.00(0)	0.1632(19)	1.00(0)	0.014(4)
**O(9)**	0.4602(9)	0.00(0)	0.1580(2)	1.00(0)	0.025(5)
**O(10)**	0.6248(7)	0.00(0)	0.1438(18)	1.00(0)	0.011(4)

Table S2. Bond distances and bond angles for the $Hg_xV_2O_5$ structure shown in Fig. 1B, obtained from Rietveld refinement of the powder XRD pattern shown in Fig. 1A (monoclinic, *C 2/m*)

V-O Polyhedra	V—O	Distance (Å)	O—V—O	Angle (°)
**$V(1)O_6$**	V(1)—O(1)	1.60(2)	O(1)—V(1)—O(2)	107.5(9)
			O(1)—V(1)—O(4)	103.6(9)
	V(1)—O(2)	1.94(2)	O(1)—V(1)—O(10)	101.7(6)
			O(1)—V(1)—O(10)	101.7(6)
	V(1)—O(4)	1.93(18)	O(4)—V(1)—O(2)	148.9(9)
			O(4)—V(1)—O(10)	98.6(6)
	V(1)—O(10)	1.90(6)	O(4)—V(1)—O(10)	98.6(7)
			O(10)—V(1)—O(2)	75.3(6)
	V(1)—O(10)	1.90(6)	O(10)—V(1)—O(2)	75.3(6)
			O(10)—V(1)—O(10)	146.7(11)
**$V(2)O_6$**	V(2)—O(3)	1.60(19)	O(3)—V(2)—O(4)	99.9(9)
			O(3)—V(2)—O(6)	102.1(9)
	V(2)—O(4)	1.94(19)	O(3)—V(2)—O(7)	105.1(5)
			O(3)—V(2)—O(7)	175.8(9)
	V(2)—O(6)	1.99(18)	O(4)—V(2)—O(6)	158.1(8)
			O(4)—V(2)—O(7)	76.0(7)
	V(2)—O(7)	1.89(4)	O(6)—V(2)—O(7)	82.1(7)
			O(7)—V(2)—O(4)	93.2(6)
	V(2)—O(7)	1.98(4)	O(7)—V(2)—O(6)	81.1(6)
			O(7)—V(2)—O(6)	158.1(8)
	V(2)—O(7)	2.38(17)	O(7)—V(2)—O(7)	147.5(10)
			O(7)—V(2)—O(7)	75.4(6)
**$V(3)O_6$**	V(3)—O(4)	2.02(18)	O(4)—V(3)—O(7)	77.1(7)
			O(5)—V(3)—O(4)	89.5(9)
	V(3)—O(5)	1.59(19)	O(5)—V(3)—O(6)	107.0(6)
			O(5)—V(3)—O(7)	166.6(9)
	V(3)—O(6)	1.90(6)	O(5)—V(2)—O(8)	100.8(9)
			O(6)—V(2)—O(4)	90.8(6)
	V(3)—O(6)	1.90(6)	O(6)—V(2)—O(6)	146.1(10)
			O(6)—V(2)—O(7)	73.7(6)
	V(3)—O(7)	2.28(19)	O(6)—V(2)—O(8)	86.2(6)
			O(8)—V(2)—O(4)	169.8(8)
	V(3)—O(8)	1.99(19)	O(8)—V(2)—O(7)	92.7(7)
**$V(4)O_5$**	V(4)—O(2)	1.91(7)	O(2)—V(4)—O(2)	143.2(12)
			O(2)—V(4)—O(10)	73.6(6)
			O(8)—V(4)—O(2)	104.0(6)
	V(4)—O(8)	1.67(19)	O(8)—V(4)— O(10)	113.8(8)
			O(9)—V(4)—O(2)	96.9(6)
	V(4)—O(9)	1.66(3)	O(9)—V(4)—O(8)	107.4(10)
			O(9)—V(4)—O(10)	138.8(9)

**Hg(1)O8**	Hg(1)—O(9)	2.13(27)	O(Hg)—V(4)—O(9)	136.6(12)

Table S3. Refinement statistics and structural details for δ-$Pb_xV_2O_5$ (Triclinic, *P-1*).

***a* = 23.4320(1) Å, *b* = 7.3974(2) Å, *c* = 8.8186(4) Å, α = 89.808(3)°, β = 88.280(5)°, γ = 89.928(2)°, V = 780.55(6) Å³, χ² = 8.66, Rp = 7.05%, wRp = 8.75%**					
**Atom**	***x***	***y***	***z***	**Occupancy**	**Uiso**
**Pb(1)**	0.2041(3)	0.0000(0)	0.0221(7)	1.011(11)	0.008(3)
**Pb(2)**	0.0458(5)	0.2514(11)	-0.0221(13)	0.496(6)	0.011(3)
**V(1)**	0.0347(14)	0.5000(0)	0.3416(4)	1.000(0)	0.007(6)
**V(2)**	0.1323(10)	0.2466(3)	0.3405(3)	1.000(0)	0.008(6)
**V(3)**	0.0299(14)	0.0000(0)	0.3408(4)	1.000(0)	0.007(6)
**V(4)**	0.1150(13)	0.5000(0)	0.6589(4)	1.000(0)	0.007(6)
**V(5)**	0.2177(10)	0.2526(3)	0.6589(3)	1.000(0)	0.008(6)
**V(6)**	0.1202(13)	0.0000(0)	0.6597(4)	1.000(0)	0.007(6)
**O(1)**	0.0212(6)	0.5000(0)	0.1633(18)	1.000(0)	0.013(3)
**O(2)**	0.0486(4)	0.7556(13)	0.3657(12)	1.000(0)	0.007(17)
**O(3)**	0.1179(6)	0.5000(0)	0.3717(16)	1.000(0)	0.008(2)
**O(4)**	0.1374(5)	0.2253(13)	0.1607(12)	1.000(0)	0.011(18)
**O(5)**	0.1174(5)	0.0000(0)	0.3986(17)	1.000(0)	0.009(2)
**O(6)**	0.0230(6)	0.0000(0)	0.1497(16)	1.000(0)	0.008(2)
**O(7)**	-0.0403(7)	0.0000(0)	0.3911(19)	1.000(0)	0.015(3)
**O(8)**	0.0449(6)	0.5000(0)	0.5965(17)	1.000(0)	0.010(3)
**O(9)**	0.2006(6)	0.5000(0)	0.6329(16)	1.000(0)	0.006(2)
**O(10)**	0.1091(7)	0.5000(0)	0.8372(18)	1.000(0)	0.014(3)
**O(11)**	0.1317(4)	0.2538(13)	0.6144(12)	1.000(0)	0.011(2)
**O(12)**	0.2927(5)	0.2512(15)	0.6020(13)	1.000(0)	0.012(2)
**O(13)**	0.2275(5)	0.2570(14)	0.8426(12)	1.000(0)	0.012(19)
**O(14)**	0.2029(6)	0.0000(0)	0.6402(16)	1.000(0)	0.008(2)
**O(15)**	0.1127(8)	0.0000(0)	0.8437(19)	1.000(0)	0.026(4)

Table S4. Bond distances and bond angles deduced for the δ-$Pb_xV_2O_5$ structure (Triclinic, *P-1*)

V-O Polyhedra	V—O	Distance (Å)	O—V—O	Angle (°)
**$V(1)O_6$**	V(1)—O(1)	1.59(16)	O(1)—V(1)—O(2)	97.8(3)
			O(1)—V(1)—O(3)	107.0(7)
	V(1)—O(2)	1.93(9)	O(2)—V(1)—O(2)	157.2(6)
			O(2)—V(1)—O(8)	97.3(3)
	V(1)—O(3)	1.96(14)	O(3)—V(1)—O(8)	79.8(3)
			O(4)—V(1)—O(10)	98.6(3)
	V(1)—O(8)	1.96(15)	O(3)—V(1)—O(8)	78.4(6)
			O(2)—V(1)—O(8)	83.1(3)
	V(1)—O(8)	2.25(15)	O(8)—V(1)—O8)	77.7(6)
			O(1)—V(1)—O(8)	174.6(7)
**$V(2)O_6$**	V(2)—O(4)	1.60(11)	O(4)—V(2)—O(12)	99.7(6)
			O(4)—V(2)—O(3)	104.9(6)
	V(2)—O(12)	1.81(11)	O(12)—V(2)—O(3)	97.2(5)
			O(12)—V(2)—O(5)	96.8(5)
	V(2)—O(3)	1.93(4)	O(3)—V(2)—O(5)	147.9(6)
			O(3)—V(2)—O(2)	79.2(5)
	V(2)—O(5)	1.93(5)	O(5)—V(2)—O(11)	75.4(5)
			O(5)—V(2)—O(12)	96.8(5)
	V(2)—O(2)	1.98(10)	O(2)—V(2)—O(4)	102.9(5)
			O(2)—V(2)—O(11)	81.0(4)
	V(2)—O(11)	2.42(11)	O(11)—V(2)—O(12)	76.3(4)
			O(11)—V(2)—O(3)	80.1(4)
**$V(3)O_6$**	V(3)—O(6)	1.69(14)	O(6)—V(3)—O(7)	101.7(7)
			O(6)—V(3)—O(2)	97.5(4)
	V(3)—O(7)	1.72(17)	O(7)—V(3)—O(2)	101.0(14)
			O(7)—V(3)—O(5)	72.3(3)
	V(3)—O(2)	1.87(9)	O(2)—V(3)—O(2)	150.1(7)
			O(2)—V(3)—O(5)	75.5(3)
	V(3)—O(2)	1.87(9)	O(2)—V(3)—O(7)	82.4(4)
			O(2)—V(3)—O(7)	82.4(4)
	V(3)—O(5)	2.09(14)	O(5)—V(3)—O(7)	72.3(6)
			O(5)—V(3)—O(2)	75.5(3)
	V(3)—O(7)	2.37(17)	O(7)—V(3)—O(5)	150.1(7)
**$V(4)O_5$**	V(4)—O(10)	1.58(16)	O(10)—V(4)—O(8)	101.4(8)
			O(10)—V(4)—O(11)	103.4(4)
	V(4)—O(8)	1.71(15)	O(8)—V(4)—O(11)	97.8(4)
			O(8)—V(4)—O(9)	154.8(7)
	V(4)—O(11)	1.91(9)	O(11)—V(4)—O(11)	145.6(6)
			O(11)—V(4)—O(9)	76.4(3)
	V(4)—O(11)	1.91(9)	O(11)—V(4)—O(9)	76.4(3)
	V(4)—O(9)	2.03(14)	O(9)—V(4)—O(10)	103.8(7)

**$V(5)O_6$**	V(5)—O(13)	1.63(11)	O(13)—V(5)—O(12)	99.9(5)
			O(13)—V(5)—O(9)	97.0(6)
	V(5)—O(12)	1.85(12)	O(12)—V(5)—O(9)	99.9(5)
			O(13)—V(5)—O(14)	97.1(6)
	V(5)—O(9)	1.89(4)	O(12)—V(5)—O(14)	98.2(6)
			O(9)—V(5)—O(14)	154.7(7)
	V(5)—O(14)	1.91(4)	O(13)—V(5)—O(11)	106.9(5)
			O(12)—V(5)—O(11)	153.2(5)
	V(5)—O(11)	2.04(11)	O(9)—V(5)—O(11)	76.6(5)
			O(14)—V(5)—O(11)	79.2(5)
	V(5)—O(12)	2.30(12)	O(13)—V(5)—O(12)	177.9(5)
			O(12)—V(5)—O(12)	78.0(5)
**$V(6)O_6$**	V(6)—O(15)	1.64(17)	O(15)—V(6)—O(7)	95.1(9)
			O(15)—V(6)—O(11)	103.0(3)
	V(6)—O(7)	1.91(16)	O(7)—V(6)—O(11)	95.4(3)
			O(7)—V(6)—O(14)	161.4(4)
	V(6)—O(11)	1.94(9)	O(11)—V(6)—O(11)	150.1(6)
			O(15)—V(6)—O(14)	103.5(8)
	V(6)—O(11)	1.94(9)	O(11)—V(6)—O(14)	80.5(3)
			O(11)—V(6)—O(5)	78.0(3)
	V(6)—O(14)	1.95(15)	O(15)—V(6)—O(5)	172.2(7)
			O(14)—V(6)—O(5)	84.3(6)
	V(6)—O(5)	2.30(15)	O(5)—V(6)—O(7)	77.1(6)
			O(5)—V(6)—O(11)	78.0(3)
**$Pb(1)O_6$**	Pb(1)—O(9)	2.55(10)	O(13)—Pb(1)—O(13)	96.5(5)
			O(13)—Pb(1)—O(15)	79.4(4)
	Pb(1)—O(13)	2.55(10)	O(13)—Pb(1)—O(4)	87.8(3)
			O(13)—Pb(1)—O(4)	155.7(3)
	Pb(1)—O(13)	2.61(18)	O(15)—Pb(1)—O(4)	77.9(4)
			O(4)—Pb(1)—O(4)	79.0(4)
	Pb(1)—O(15)	2.62(10)	O(13)—Pb(1)—O(13)	130.0(14)
			O(13)—Pb(1)—O(13)	68.5(4)
	Pb(1)—O(4)	2.62(10)	O(15)—Pb(1)—O(13)	137.5(2)
			O(4)—Pb(1)—O(13)	125.5(3)
	Pb(1)—O(13)	2.66(11)	O(13)—Pb(1)—O(13)	68.5(4)
			O(13)—Pb(1)—O(15)	137.5(2)
	Pb(1)—O(13)	2.66(11)	O(13)—Pb(1)—O(4)	125.5(3)
			O(13)—Pb(1)—O(13)	85.0(5)
**$Pb(2)O_6$**	Pb(2)—O(1)	2.54(11)	O(1)—Pb(2)—O(6)	95.2(4)
	Pb(2)—O(1)	2.69(11)	O(1)—Pb(2)—O(4)	82.0(4)
	Pb(2)—O(4)	2.64(11)	O(4)—Pb(2)—O(6)	131.8(3)
	Pb(2)—O(6)	2.47(9)	O(6)—Pb(2)—O(1)	131.4(4)
	Pb(2)—O(6)	2.68(10)	O(6)—Pb(2)—O(10)	158.8(5)
	Pb(2)—O(10)	2.70(11)	O(10)—Pb(2)—O(15)	85.9(4)

	Pb(2)—O(15)	2.73(13)	O(15)—Pb(2)—O(6)	84.0(4)

Table S5. ***Crystal data and structure refinement for δ-$Tl_{0.5}V_2O_5$: TlV2O5_6601_0m_a. CSD deposition number: 2170921***

**Identification code**	TlV2O5_6601_0m_a	
**Empirical formula**	O5 Tl0.51 V2	
**Formula weight**	286.65	
**Temperature**	110(2) K	
**Wavelength**	0.71073 Å	
**Crystal system**	Monoclinic	
**Space group**	C 2/m	
**Unit cell dimensions**	*a* = 11.538(3) Å	α= 90°.
	b = 3.6917(10) Å	β= 100.779(11)°.
	c = 9.527(3) Å	ɣ = 90°.
**Volume**	398.65(19) $Å^3$	
**Z**	4	
**Density (calculated)**	4.776 mg/m3	
**Absorption coefficient**	25.205 mm-1	
**F(000)**	510	
**Crystal size**	0.085 × 0.045 × 0.005 mm^3	
**Theta range for data collection**	2.176 to 30.985°.	
**Index ranges**	-16<=h<=16, -5<=k<=5, -13<=l<=13	
**Reflections collected**	5031	
**Independent reflections**	728 [R(int) = 0.0677]	
**Completeness to theta = 25.242°**	99.50%	
**Absorption correction**	Semi-empirical from equivalents	
**Max. and min. transmission**	0.746 and 0.563	
**Refinement method**	Full-matrix least-squares on F2	
**Data / restraints / parameters**	728 / 0 / 54	
**Goodness-of-fit on F^2**	1.133	
**Final R indices [I>2sigma(I)]**	R1 = 0.0338, wR2 = 0.0617	
**R indices (all data)**	R1 = 0.0369, wR2 = 0.0628	
**Largest diff. peak and hole**	1.908 and -1.791 e.Å-3	

Table S6. ***Atomic coordinates (x 10^4), occupancies, and equivalent isotropic displacement parameters ($Å^2$x 10^3) for δ-$Tl_{0.5}V_2O_5$: TlV2O5_6601_0m_a. U(eq) is defined as one third of the trace of the orthogonalized U^{ij} tensor.***

Atom	*x*	*y*	*z*	Occupancy	Uiso
Tl(1)	5000	10000	5000	1	13(1)
Tl(2)	3040(30)	5000	4990(30)	0.013	19(9)
V(1)	2923(1)	5000	1569(1)	1	9(1)
V(2)	5927(1)	5000	1554(1)	1	10(1)
O(1)	2591(5)	0	1143(6)	1	9(1)
O(2)	3349(5)	5000	3269(6)	1	11(1)
O(3)	4335(5)	5000	947(6)	1	9(1)
O(4)	6042(5)	5000	3269(6)	1	12(1)
O(5)	6184(5)	0	1286(6)	1	10(1)

Table S7. ***Anisotropic displacement parameters ($Å^2$x 10^3) for δ-$Tl_{0.5}V_2O_5$: TlV2O5_6601_0m_a. The anisotropic displacement factor exponent takes the form: -2□^{2}[h^2 $a^{*2}U^{11}$ + ... + 2 h k a* b* U^{12}]***

Atom	U11	U22	U33	U23	U13	U12
Tl(1)	20(1)	7(1)	12(1)	0	1(1)	0
Tl(2)	30(17)	17(13)	9(13)	0	1(10)	0
V(1)	11(1)	7(1)	9(1)	0	0(1)	0
V(2)	14(1)	7(1)	9(1)	0	4(1)	0
O(1)	11(3)	7(2)	11(3)	0	5(2)	0
O(2)	13(3)	16(3)	6(2)	0	3(2)	0
O(3)	7(2)	8(2)	11(3)	0	2(2)	0
O(4)	14(3)	16(3)	7(3)	0	2(2)	0
O(5)	11(3)	6(2)	12(3)	0	3(2)	0

Table S8. Bond distances and bond angles for the structure shown in Fig. 1D, obtained from *the refinement of single crystal data of δ-$Tl_xV_2O_5$*.

V-O Polyhedra	V—O	Distance (Å)	O—V—O	Angle (°)
**$V(1)O_6$**	V(1)—O(1)	1.913(3)	O(2)—V(1)—O(3)	101.7(3)
	V(1)—O(1)	1.913(3)	O(2)—V(1)—O(1)	103.1(18)
	V(1)—O(2)	1.602(13)	O(3)—V(1)— O(1)	95.0(18)
			O(1)—V(1)—O(1)	149.5(3)
	V(1)—O(3)	1.836(16)	O(2)—V(1)—O(5)	104.5(3)
			O(3)—V(1)—O(5)	153.8(3)
	V(1)—O(4)	2.541(10)	O(1)—V(1)—O(5)	79.2(18)
			O(2)—V(1)—O(1)	103.1(18)
	V(1)—O(5)	1.975(14)	O(3)—V(1)—O(1)	95.1(18)
			O(1)—V(1)—O(5)	79.2(18)
**$V(2)O_6$**	V(2)—O(1)	1.030(7)	O(4)—V(2)—O(3)	102.1(3)
	V(2)—O(3)	1.818(6)	O(4)—V(2)—O(5)	98.7(19)
	V(2)—O(3)	2.245(6)	O(3)—V(2)—O(5)	97.5(19)
	V(2)—O(4)	1.617(6)	O(5)—V(2)—O(5)	154.0(4)
	V(2)—O(5)	1.894(16)	O(4)—V(2)—O(1)	107.0(3)
			O(3)—V(2)—O(1)	150.9(3)
	V(2)—O(5)	1.894(16)	O(5)—V(2)—O(1)	78.3(3)
			O(4)—V(2)—O(3)	177.4(3)
**$Tl(1)O_8$**	Tl(2)—O(2)	1.740(3)	O(2)—Tl(2)—O(4)	133.0(19)
	Tl(2)—O(4)	1.790(3)		

Table S9. Selected V—V bond distances for $M_xV_2O_5$ (M = Hg, Tl, Pb)

	V—V distances (Å)
**$Hg_{0.5}V_2O_5$**	
V1—V3	3.076(6)
V1—V4	3.084(5)
V2—V3	3.120(5)
V2—V3	3.347(6)
V2—V2	3.395(7)
**δ-$Tl_{0.5}V_2O_5$**	
V1—V1	3.688(2)
V1—V1	3.492(4)
V1—V2	2.992(5)
V1—V2	3.448(5)
V2—V2	3.265(6)
**δ-$Pb_{0.5}V_2O_5$**	
V1—V1	3.288(8)
V1—V3	3.700(5)
V4—V5	3.022(4)
V5—V6	2.951(4)

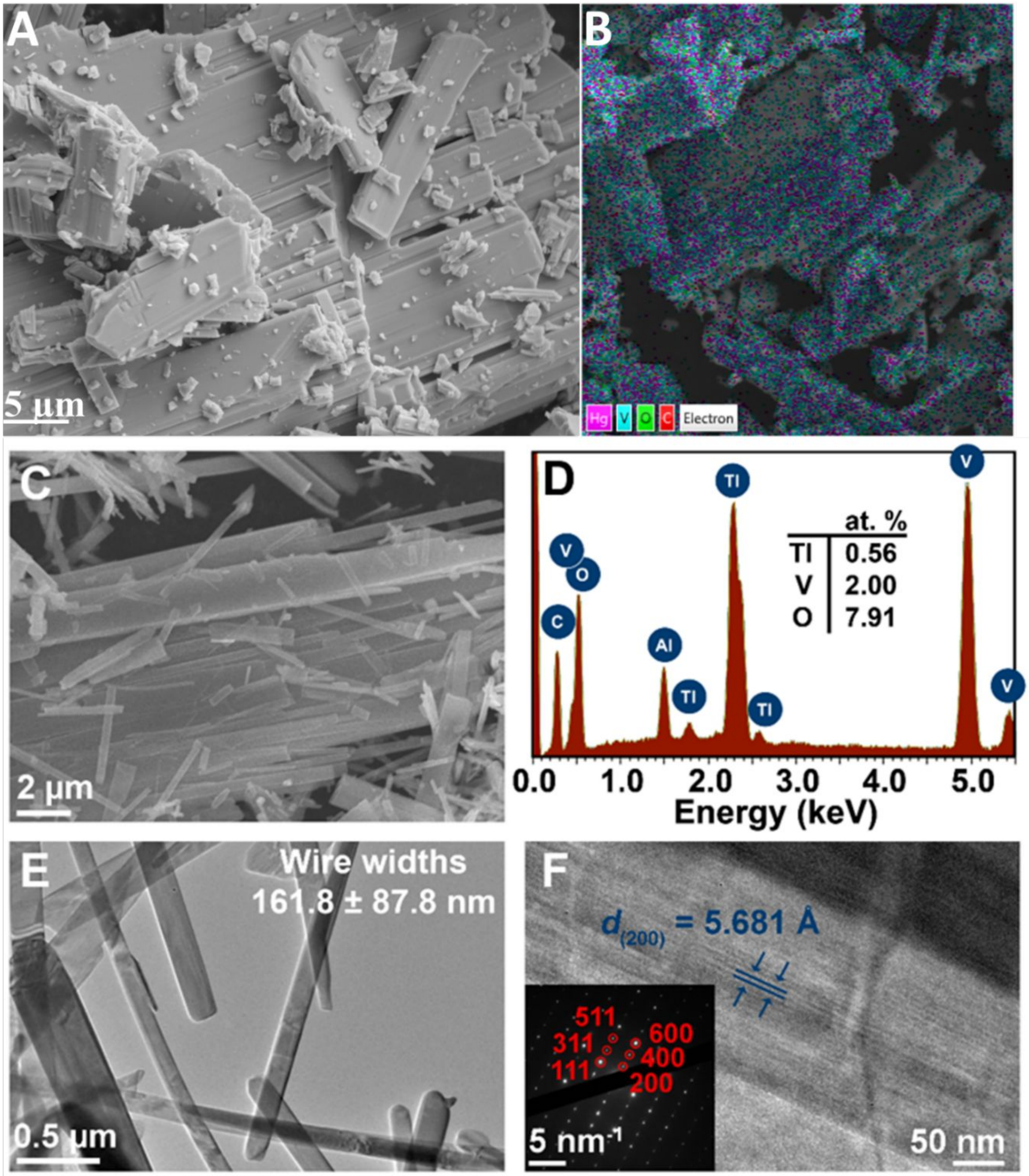

Figure S1. Electron microscopy characterization of δ-$Tl_xV_2O_5$ and $Hg_xV_2O_5$. *(A) Scanning electron microscopy (SEM) image of $Hg_xV_2O_5$ particles with lengths spanning hundreds of micrometers. (B) Energy dispersive X-ray spectroscopy (EDS) map of the elemental distributions of Hg, V, and O. (C) SEM image of δ-$Tl_xV_2O_5$ nanowires. (D) EDS integrated across δ-$Tl_xV_2O_5$ nanowires shown in (C). (E) Low-magnification transmission electron microscopy (TEM) image of δ-$Tl_xV_2O_5$ nanowires showing an average width of 162±88 nm. (F) Lattice-resolved TEM image with selected area electron diffraction (SAED) pattern indexed to the C 2/m cell derived from Rietveld refinement of the powder XRD pattern.*

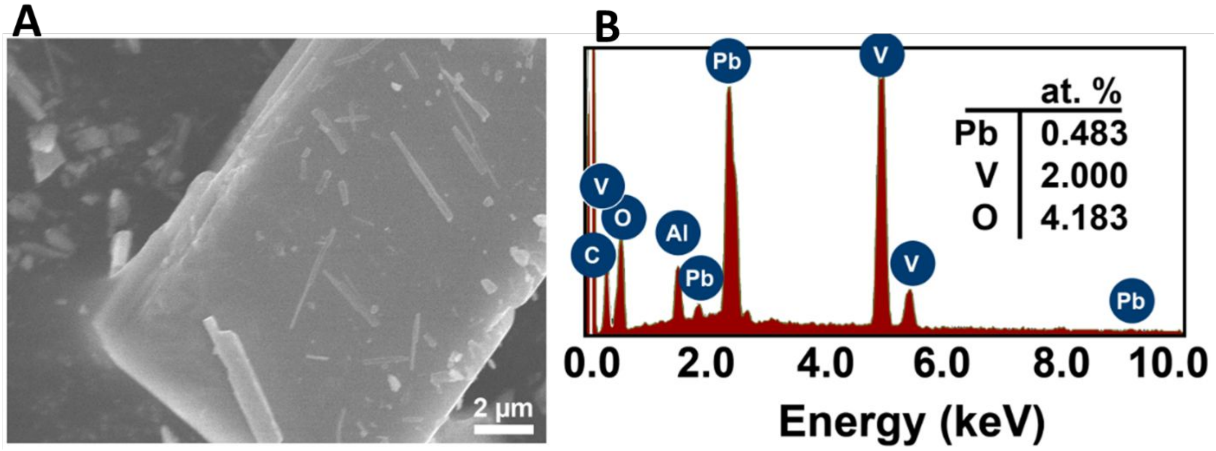

Figure S2. Electron microscopy characterization of *δ-$Pb_xV_2O_5$*. *(A) SEM image collected for a large δ-$Pb_xV_2O_5$ crystal and (B) corresponding EDS spectrum corroborating the assigned stoichiometry of δ-$Pb_{0.5}V_2O_5$.*

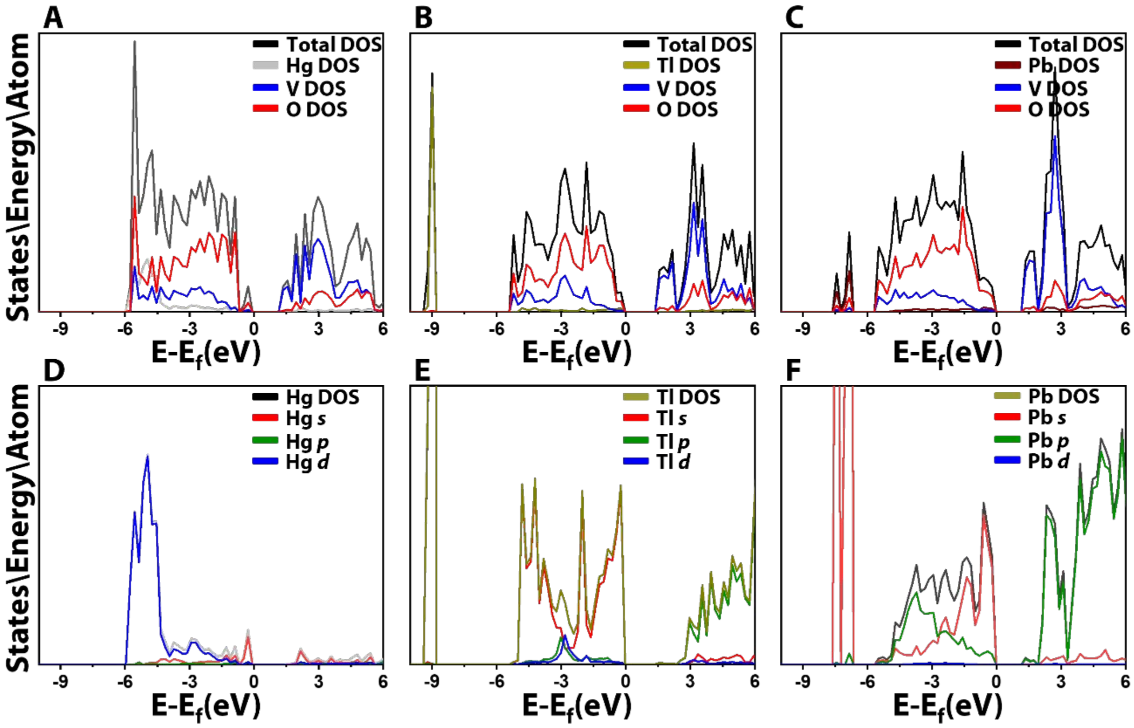

Figure S3. Density of States as calculated from ground state electronic structure calculations. *(A) Total and atom-projected density of states for $Hg_{0.5}V_2O_5$. (B) Total and atom-projected density of states for δ-$Tl_{0.5}V_2O_5$ (C) Total and atom-projected density of states for δ-$Pb_{0.5}V_2O_5$. (D) Orbital-projected density of states for Hg 5d and 6s orbitals in $Hg_{0.5}V_2O_5$. (E) Orbital--projected density of states for Tl in δ-$Tl_{0.5}V_2O_5$. (F) Orbital projected density of states for Pb in δ-$Pb_{0.5}V_2O_5$.*

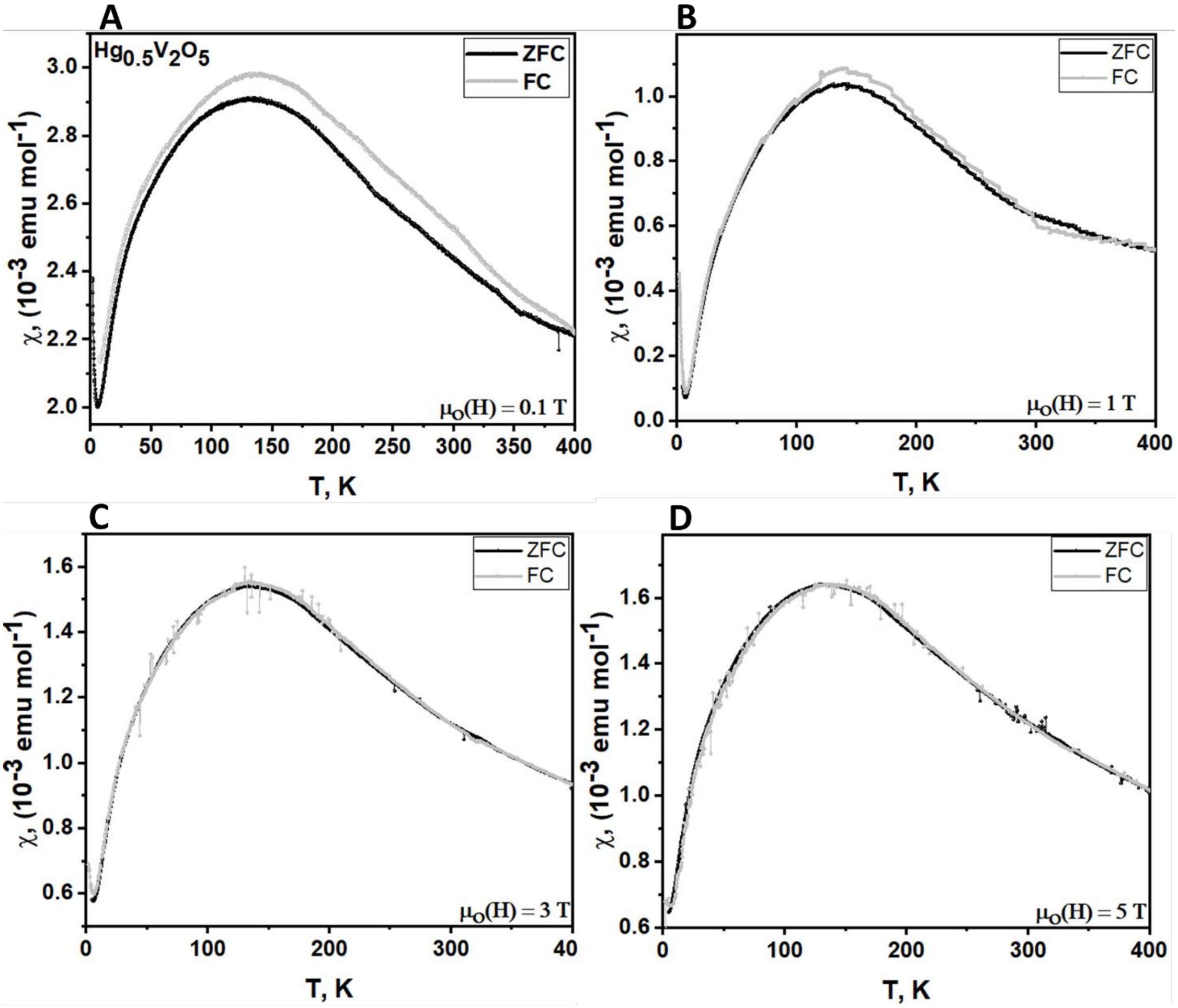

Figure S4**. **Magnetic susceptibility measurements of $Hg_{0.5}V_2O_5$ at varying field strengths. *Temperature dependence of magnetic susceptibility of $Hg_{0.5}V_2O_5$ between 2−400K at an applied field of (A) 0.1 T, (B) 1 T, (C) 3 T, and (D) 5 T.*

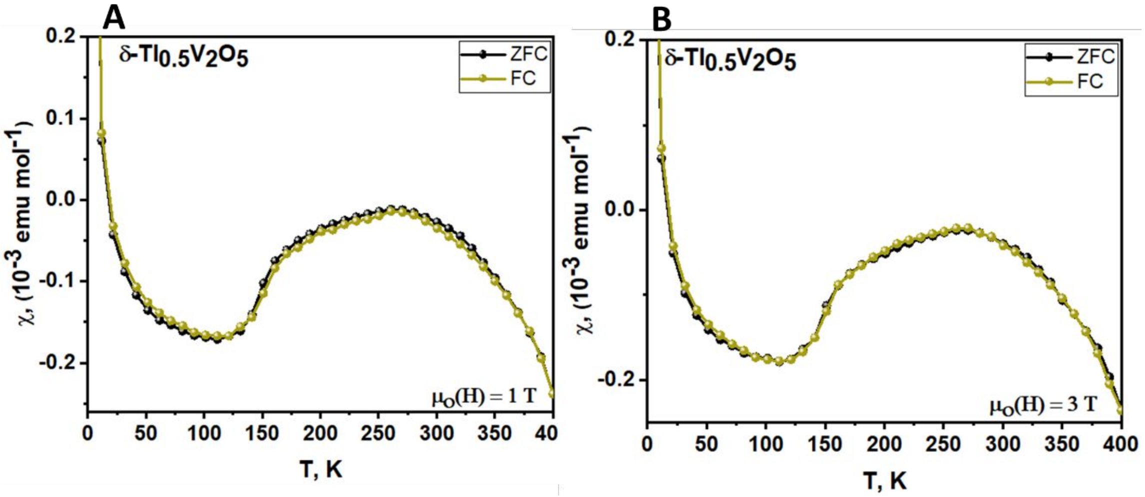

***Figure S5*. Temperature dependence of Magnetic susceptibility of δ-$Tl_{0.5}V_2O_5$ at different applied fields.** *ZFC and FC magnetic susceptibility curves of δ-$Tl_{0.5}V_2O_5$ at an applied field of (A) 1 T and (B) 3 T.*

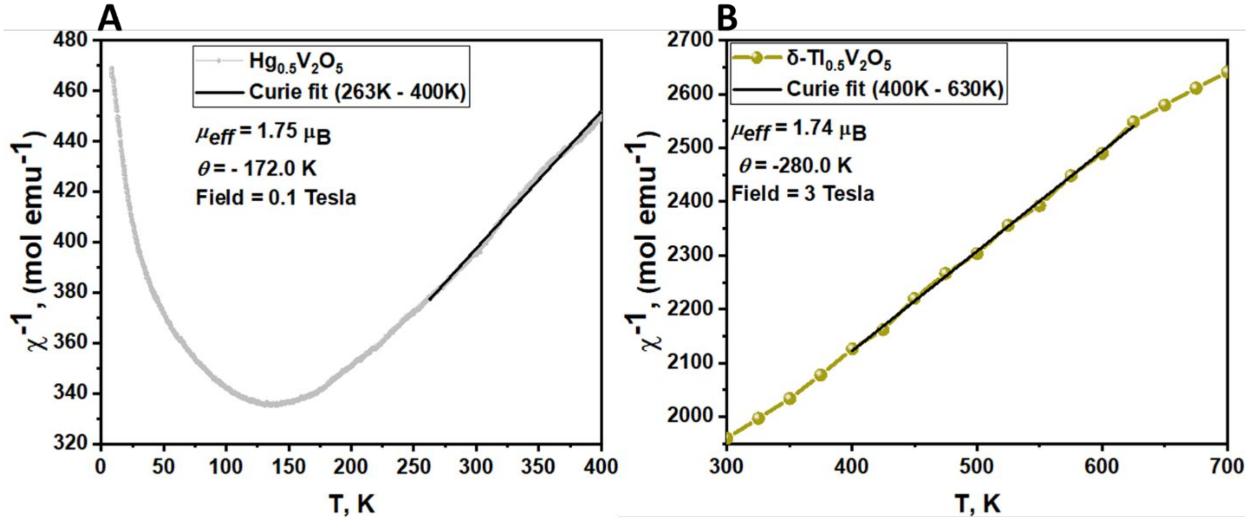

***Figure S6*. Inverse magnetic susceptibility of $Hg_xV_2O_5$ and δ-$Tl_xV_2O_5$.** *Reciprocal plot of the magnetic susceptibility data at 2−400 K and 300—700 K and results of the Curie−Weiss fitting for (A) $Hg_{0.5}V_2O_5$ and (B) δ-$Tl_{0.5}V_2O_5$, respectively. (For δ-$Tl_{0.5}V_2O_5$, the compound exhibits a nonlinear dependence of inverse susceptibility at low temperatures (between 280-400 K), making accurate determination of valid Weiss constants or effective moments difficult. As such, we collected data well above the transition temperature (400-700 K)).*

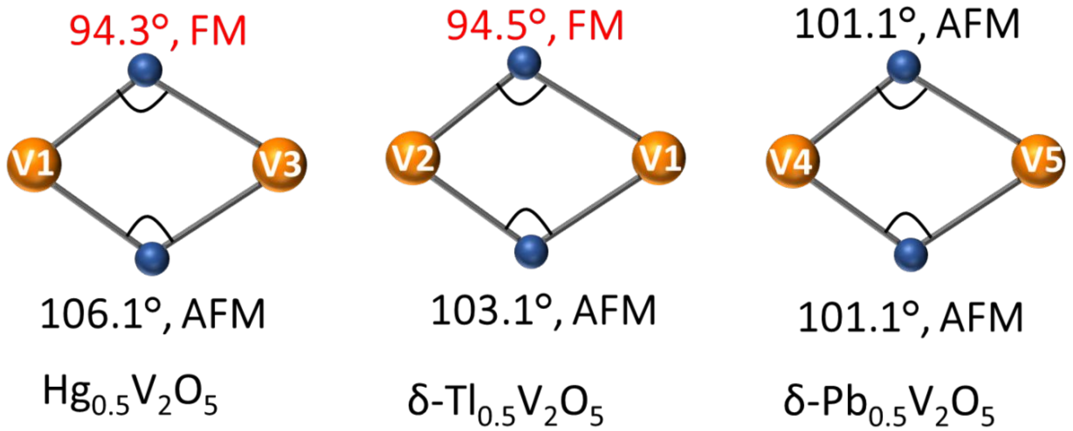

Figure S7. Principal superexchange interaction as a function of the V—O—V bond angles (°) in (left) *$Hg_{0.5}V_2O_5$, (**middle**) δ-$Tl_{0.5}V_2O_5$, and (**right**) δ-$Pb_{0.5}V_2O_5$. If a cation-anion-cation bond angle is nearly 90◦, the system prefers FM ordering; conversely, the system prefers AFM ordering for bond angles approaching 180◦.*
